# Genetic subtype-guided immunochemotherapy in relapsed and refractory diffuse large B cell lymphoma: a phase 2 investigator-initiated nonrandomized clinical trial (GUIDANCE-06)

**DOI:** 10.1038/s41392-025-02316-6

**Published:** 2025-07-26

**Authors:** Yi-Ge Shen, Qing Shi, Wei Tang, Peng-Peng Xu, Yi-Wen Cao, Meng-Meng Ji, Zhong Zheng, Shu Cheng, Li Wang, Wei-Li Zhao

**Affiliations:** 1 Shanghai Institute of Hematology, State Key Laboratory of Medical Genomics; National Research Center for Translational Medicine at Shanghai, Ruijin Hospital Affiliated to Shanghai Jiao Tong University School of Medicine, Shanghai, China; 2 Pôle de Recherches Sino-Français en Science du Vivant et Génomique, Laboratory of Molecular Pathology, Shanghai, China

**Keywords:** Drug development, Cancer genetics, Cancer microenvironment

## Abstract

Improving the outcome of relapsed or refractory diffuse large B-cell lymphoma (R/R DLBCL) remained an unmet need. The aim of this single-center, phase 2 trial was to evaluate the efficacy and safety of genetic subtype-guided immunochemotherapy (R-ICE-X) in patients with R/R DLBCL: R-ICE-zanubrutinib for MCD-like and BN2-like, R-ICE-lenalidomide for N1-like and NOS, R-ICE-decitabine for *TP53*^Mut^, R-ICE-chidamide for EZB-like, and R-ICE-tofacitinib for ST2-like subtype. Enrolled patients were treated with assigned regimens for three cycles, and then responders were treated with autologous hematopoietic stem cell transplantation (ASCT) or 3 cycles of R-ICE-X consolidation and lenalidomide maintenance. The primary endpoint was the complete response (CR) rate. The secondary endpoints included overall response rate (ORR), progression-free survival (PFS), overall survival (OS), and safety assessment. Between April 26, 2022, and July 31, 2024, 76 patients were enrolled, with 74 adhering to and 2 deviating from the protocol. Among all, the CR rate was 56.6% (95% CI, 45.2–68.0%), and the ORR was 76.3% (95% CI, 66.5–86.1%) at the end of induction. With a median follow-up of 19.5 months, the 2-year PFS rate was 69.3% (95% CI, 56.6–79.0%), and the 2-year OS rate was 88.3% (95% CI, 77.6–94.0%). The primary grade 3-4 adverse events were neutropenia (30%) and thrombocytopenia (25%). The presence of bulky disease and *CD70* mutation was linked to poor prognosis. Further gene set enrichment analysis revealed that up-regulated PI3K-AKT-mTOR signaling pathway and reduced immune cell infiltration were significantly associated with disease progression. Patients with mesenchymal or inflammatory lymphoma microenvironment subtypes benefited from R-ICE-X treatment. Our findings highlight the efficacy and safety of R-ICE-X, a mechanism-based tailored therapy, which dually targets genetic and microenvironmental alterations in R/R DLBCL.

## Introduction

Diffuse large B-cell lymphoma (DLBCL), the most prevalent aggressive non-Hodgkin lymphoma subtype, exhibits marked heterogeneity in clinical behavior and molecular pathogenesis.^1^ Rituximab plus cyclophosphamide, doxorubicin, vincristine, and prednisone (R-CHOP) regimen remains the therapeutic mainstay for newly diagnosed cases, inducing remission in about 60% of patients.^2^ Nevertheless, when stratified by cumulative adverse prognostic parameters in the International Prognostic Index (IPI), 20–50% of patients with DLBCL exhibit primary refractoriness to R-CHOP immunochemotherapy or experience relapse following first-line remission.^3^ Refractory or relapsed (R/R) DLBCL carries a dismal prognosis, with a median survival of less than 12 months for chemotherapy-resistant disease. While high-dose chemotherapy followed by autologous stem cell transplantation (ASCT) offers a potential cure for chemosensitive patients,^4^ its clinical utility remains limited by the absence of salvage immunochemotherapy regimens that consistently achieve deep molecular responses while maintaining adequate tolerability. Emerging insights from multi-omics profiling have unveiled distinct molecular mechanisms within DLBCL, including dysregulated B-cell receptor (BCR) signaling, epigenetic reprogramming, and immune checkpoint dysregulation, necessitating a shift from histomorphology-driven to genotype-guided therapeutic strategies.

Conventional salvage approaches including ICE (ifosfamide, carboplatin, etoposide), DHAP (dexamethasone, high-dose cytarabine, cisplatin), or GDP (gemcitabine, dexamethasone, cisplatin), demonstrate comparable efficacy and toxicity profiles, with no regimen proving definitively superior.^5,6^ Among these, the R-ICE regimen (rituximab plus ICE) remains a cornerstone salvage therapy for R/R DLBCL. Phase III data from the CORAL trial (NCT00137995) reveal modest overall long-term efficacy, with a complete response (CR) rate of 24.4% and 3-year progression-free survival (PFS) of 31%, underscoring new combinatorial strategies in high-risk subsets to overcome potential disease relapse driven by clonal evolution and microenvironmental alterations.^6^ Given that the cure rate of the conventional second-line therapy is suboptimal, extensive efforts to optimize salvage immunochemotherapy have explored regimen intensification strategies, including rituximab dose escalation and radioimmunotherapy consolidation, yet failed to demonstrate clinically meaningful improvements.^7,8^ More recently, technological advances in high-throughput sequencing have catalyzed a paradigm shift in deciphering the molecular pathogenesis of DLBCL and facilitated the establishment of genetic classification models. Among them, the simplified LymphPlex algorithm displayed high efficacy and clinical practicability, contributing to optimizing mechanism-based immunochemotherapy for newly diagnosed DLBCL.^9^ Based on targeted sequencing and fluorescence in situ hybridization (FISH), LymphPlex classified DLBCL into seven genetic subtypes with distinct clinicopathological characteristics and prognosis, including MCD-like (characterized by *MYD88*/*CD79B* mutations), BN2-like (*NOTCH2/BCL6* alterations), *TP53*^Mut^ (*TP53* mutations), EZB-like (*BCL2/EZH2* aberrations), ST2-like (JAK-STAT activation), N1-like (*NOTCH1* mutations), and not otherwise specified (NOS). Each subtype exhibits distinct clinicopathological features, treatment responses, and survival outcomes, challenging the continued reliance on therapeutic strategy using a unified regimen.

Building upon the precision oncology paradigm, the phase 2 GUIDANCE-01 trial established genotype-guided immunochemotherapy (R-CHOP-X) as a frontline strategy for newly diagnosed DLBCL. The trial met its primary endpoint, with R-CHOP-X demonstrating statistically significant improvements over standard R-CHOP in response rates, alongside superior survival outcomes. These research results were encouraging, confirming the therapeutic imperative of molecularly informed combinatorial strategies to counteract subtype-specific resistance pathways.^10^ Translating this precision medicine approach to salvage therapy, we designed different targeted agents combined with the R-ICE regimen guided by genetic subtypes for R/R DLBCL. Zanubrutinib, an oral Bruton tyrosine kinase inhibitor (BTKi), demonstrates anti-tumor activity in R/R DLBCL, particularly in MCD-like patients harboring *MYD88*/*CD79B* co-mutations.^11^ Zanubrutinib also selectively antagonizes the tonic BCR signaling pathway, thereby abrogating the constitutive NF-κB activation that defines the molecular pathogenesis of the BN2-like DLBCL subtype.^12^ Therefore, therapeutic integration of zanubrutinib was prioritized for the MCD-like and BN2-like subtypes. As demonstrated in our prior mechanistic studies, epigenetic modulation via decitabine enhances chemotherapy sensitivity through Th1-mediated immune potentiation,^13^ supporting the addition of decitabine to the *TP53*^Mut^ subtype. The EZB-like subtype is driven by *BCL2* gene rearrangement and *EZH2* mutations. Chidamide, a histone deacetylase inhibitor (HDACi), suppresses the levels of EZH2 and exerts anti-tumor activity,^14^ which could show benefits in the EZB-like subtype. The ST2-like subtype with constitutive Janus kinase or signal transducers and activators of transcription (JAK-STAT) signaling pathway could respond to the pan-JAK inhibitor tofacitinib, which suppresses STAT3-driven immunosuppressive cytokine production.^15^ Lenalidomide, a cereblon-targeting immunomodulatory agent (IMiD), exerts multimodal immunotherapeutic effects through T-cell co-stimulation, natural killer (NK) cell cytotoxicity enhancement, and suppression of protumoral cytokines.^16^ In patients with the N1-like subtype and NOS who exhibit limited biological features, lenalidomide-based regimens may demonstrate salvage potential by counterbalancing the immunosuppressive microenvironment.

This phase 2 clinical trial (GUIDANCE-06) prospectively evaluated the clinical integration of molecularly defined targeted agents in combination with R-ICE (R-ICE-X) for R/R DLBCL. The primary objectives were to assess clinical outcomes, including therapeutic efficacy and safety profiles. Meanwhile, secondary analyses focused on identifying predictive biomarkers through comprehensive genomic profiling and characterizing tumor immune microenvironment dynamics associated with differential treatment responses. This study represents the first systematic effort to apply molecular classification in salvage therapy while dissecting bidirectional crosstalk between genetic driver mutations and tumor immune microenvironment, which was a critical step toward mechanism-based personalized management of high-risk R/R DLBCL.

## Results

### Patients and treatment

Between April 26, 2022, and July 31, 2024, the therapeutic cohort consisted of 76 prospectively enrolled evaluable subjects as the intent-to-treat (ITT) population for efficacy and safety analysis. Of these, 32 (42%) were men and 44 (58%) were women, with a median age of 61 years (range, 18–71 years). The baseline characteristics like age, sex, disease status, Eastern Cooperative Oncology Group (ECOG) performance status, serum lactate dehydrogenase (LDH), Ann Arbor stage, extranodal involvement, bulky disease, systemic symptoms, IPI score, and pathological features were summarized in Table 1 and Supplementary Table 1. Most patients presented high-risk clinical-pathological features at enrollment: 60 (79%) with serum LDH elevation exceeding institutional thresholds, 55 (72%) with Ann Arbor stage III/IV disease, 34 (45%) with multifocal extranodal involvement (≥2 sites), and 64 (84%) with intermediate-risk or high-risk IPI categories. Immunohistochemical analysis revealed a non-germinal center B-cell-like (non-GCB) cell-of-origin subtype in 70% of cases, concurrent with MYC/BCL2 dual protein co-expression in 47% of cases. Three (4%) patients harbored *MYC* and *BCL6* rearrangements, while none exhibited high-grade B-cell lymphoma with *MYC* and *BCL2* and/or *BCL6* rearrangements. Seventy-one (93%) cases received R-ICE-X as second-line therapy, and 5 (7%) received it as third- or later-line therapy.

**Table 1:** Baseline characteristics of patients with R/R DLBCL who received R-ICE-X

Characteristic, N(%)	ALL (n = 76)	R-ICE-zanubrutinib (n = 34)	R-ICE-lenalidomide (n = 30)	R-ICE-decitabine (n = 9)
Age at enrollment, years				
Median (range), y	61 (18–71)	62 (18–66)	61 (18–71)	60 (41–64)
Disease status				
Refractory	39 (51)	16 (47)	12 (40)	8 (89)
Relapsed	37 (49)	18 (53)	18 (60)	1 (11)
Gender				
Female	44 (58)	16 (47)	17 (57)	6 (67)
Male	32 (42)	18 (53)	13 (43)	3 (33)
ECOG performance status				
0–1	67 (88)	30 (88)	26 (87)	8 (89)
2	9 (12)	4 (12)	4 (13)	1 (11)
Lactic dehydrogenase				
Normal	16 (21)	7 (21)	9 (30)	0 (0)
Elevated	60 (79)	27 (79)	21 (70)	9 (100)
Ann Arbor stage				
I-II	21 (28)	11 (32)	8 (27)	2 (22)
III-IV	55 (72)	23 (68)	22 (73)	7 (78)
Extranodal involvement				
0–1 site	42 (55)	18 (53)	16 (53)	6 (67)
≥2 sites	34 (45)	16 (47)	14 (47)	3 (33)
Bulky disease				
No	64 (84)	28 (82)	26 (87)	7 (78)
Yes	12 (16)	6 (18)	4 (13)	2 (22)
Systemic symptoms				
Absence	28 (37)	12 (35)	10 (33)	4 (44)
Presence	48 (63)	22 (65)	20 (67)	5 (56)
International Prognostic Index				
Low risk	12 (16)	5 (15)	6 (20)	5 (56)
Intermediate risk	42 (55)	20 (59)	14 (47)	1 (11)
High risk	22 (29)	9 (26)	10 (33)	3 (33)
Cell of origin				
Germinal center B-cell	23 (30)	11 (32)	8 (27)	2 (22)
Non-germinal center B-cell	53 (70)	23 (68)	22 (73)	7 (78)
MYC and BCL2 or BCL6 re-arrangements				
No	76 (100)	34 (100)	30 (100)	9 (100)
Yes	0 (0)	0 (0)	0 (0)	0 (0)
BCL2-MYC double expression				
No	40 (53)	17 (50)	17 (57)	5 (56)
Yes	36 (47)	17 (50)	13 (43)	4 (44)
Prior treatment				
1 line	71 (93)	31 (91)	28 (93)	9 (100)
≥2 lines	5 (7)	3 (9)	2 (7)	0 (0)

Data are n (%)

*ECOG* Eastern Cooperative Oncology Group, *R-ICE* rituximab plus ifosfamide, carboplatin and etoposide

**Fig. 1:**
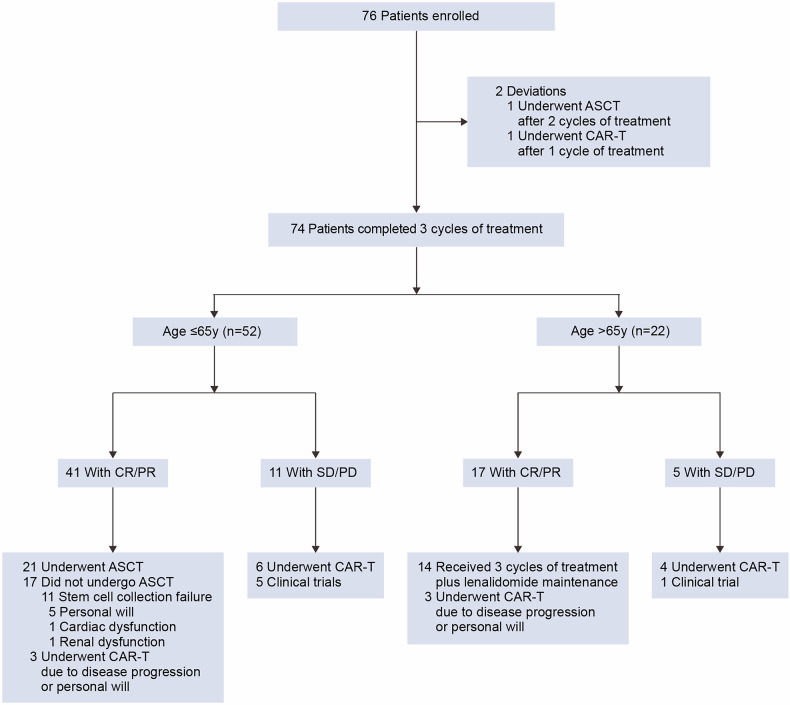
Study flow diagram. *R-ICE-X* rituximab, ifosfamide, *CR* carboplatin and etoposide combined with targeted agents. complete response, *PR* partial response, *SD* stable disease, *PD* progressive disease, *ASCT* autologous stem cell transplantation, *CAR-T* chimeric antigen receptor T-cell therapy

Following initial R-ICE induction therapy, all participants underwent genotype-guided therapeutic stratification, transitioning to the R-ICE-X regimen for subsequent treatment cycles. Seventy-four (97%) patients completed the three cycles of treatment, while one patient underwent chimeric antigen receptor (CAR)-T cell therapy after one cycle of R-ICE-zanubrutinib, and another patient underwent ASCT consolidation after two cycles of R-ICE-lenalidomide (Fig. 1). Among the 74 patients, 52 (70%) patients were aged 65 years or younger, and 21 of the 41 achieving remission underwent ASCT. The other 20 patients did not receive ASCT due to the failed stem cell mobilization (n = 11), patient preference (n = 5), other severe diseases (n = 2, 1 with cardiac dysfunction and 1 with renal dysfunction), or CAR-T cell therapy (n = 3). Fourteen (64%) of 22 patients ineligible for ASCT completed 6 cycles of induction and initiated lenalidomide maintenance. Sixteen of 74 patients (22%) received CAR-T cell therapy, including 12 due to rapid disease progression and 4 due to patient preference after remission.

### Efficacy measures

**Fig. 2:**
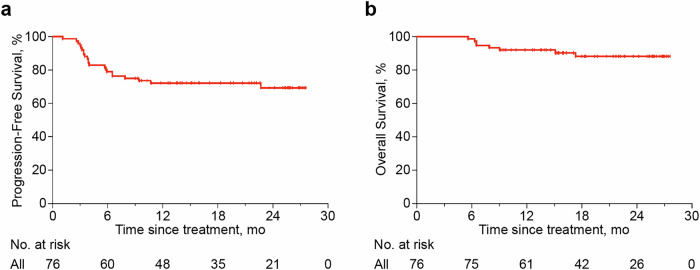
Progression-free survival and overall survival of patients with R/R DLBCL who received R-ICE-X. **a** Progression-free survival of the entire cohort. **b** Overall survival of the entire cohort. *R-ICE-X* rituximab, ifosfamide, carboplatin, and etoposide combined with targeted agents

The primary endpoint of the CR rate was 56.6% (43 patients, 95% CI 45.2–68.0%), and the overall response rate (ORR) was 76.3% (58 patients, 95% CI 66.5–86.1%). Disease status assessments identified stable disease (SD) in three (4%) patients and progressive disease (PD) in 13 (17%) patients. At a median follow-up of 19.5 months, the 2-year PFS rate was 69.3% (95% CI 56.6–79.0%) and the 2-year overall survival (OS) rate was 88.3% (95% CI 77.6–94.0%; Fig. 2a, b). Eight (11%) deaths occurred, primarily due to post-ASCT or post-CAR-T complications in 5 patients. The other three patients died from rapid disease progression. No fatal adverse events were attributed to treatment.

Genetic clustering using the LymphPlex algorithm defined seven subgroups: MCD-like (25%), BN2-like (20%), N1-like (5%), NOS (34%), *TP53*^Mut^ (12%), EZB-like (3%), and ST2-like (1%) subtypes. Subgroup analyses showed that the ORR was 79.4% in the R-ICE-zanubrutinib cohort, with 18 patients (53%) achieving CR and 9 patients (26%) achieving partial response (PR). Corresponding 2-year PFS and OS were 70.2% and 80.3%, respectively. No significant differences were apparent between MCD-like and BN2-like subtypes (Supplementary Fig. 1). In the R-ICE-lenalidomide cohort, 17 patients (57%) achieved CR, and 6 patients (20%) achieved PR. Corresponding 2-year PFS and OS were 73.3% and 100.0%, respectively. No significant differences were apparent between N1-like and NOS subtypes (Supplementary Fig. 2). In the R-ICE-decitabine group, 6 patients (67%) achieved CR. Longitudinal survival metrics revealed 2-year PFS of 55.6% and OS of 77.8%, respectively (Supplementary Fig. 3). Two patients in the R-ICE-chidamide group remained in continuous remission. One patient in the R-ICE-tofacitinib group presented disease progression. Univariate analysis indicated bulky disease as a negative prognostic factor, while cell-of-origin and MYC/BCL2 co-expression lacked statistical significance (Supplementary Table 2). Among 21 ASCT recipients, the 2-year PFS and OS rates were both at 90.5%, comparable to non-ASCT patients (Supplementary Fig. 4).

### Safety

Hematologic toxicity predominated in the safety analysis, with cytopenia being the most frequent treatment-emergent adverse event (TEAE). Neutropenia occurred in 60 (79%) patients, with grade 3–4 severity observed in 23 (30%). Thrombocytopenia developed in 52 (68%) patients, and grade 3-4 severity occurred in 19 (25%) patients without treatment-related bleeding complications. Anemia developed in thirty-nine (51%) patients, while grade 3-4 anemia was restricted to 4 (5%). For non-hematological toxicities, nausea and vomiting were reported in 7 (9%) patients without grade ≥3 occurrences. Increased alanine transaminase (ALT) level was reported in 20 (26%) patients, including two grade 3-4 cases. Increased aspartate transaminase (AST) level was observed in 18 (24%) patients, including one grade 3-4 event. Oral mucositis was documented in 3 (4%) patients. Infection occurred in 7 (9%) patients, including five grade 3-4 events. Treatment-related neurotoxicity occurred in 4 (5%) patients, with no cardiotoxicity reported. TEAE frequencies across treatment arms were summarized in Table 2.

**Table 2:** Adverse events

	All patients		R-ICE-zanubrutinib (n = 34)		ICE-lenalidomide (n = 30)		R-ICE-decitabine (n = 9)	
Adverse events	Any grade	Grade ≥3^a^	Any grade	Grade ≥3^a^	Any grade	Grade ≥3^a^	Any grade	Grade ≥3^a^
**Hematological events**								
Neutropenia	60 (79)	23 (30)	27 (79)	10 (29)	24 (80)	9 (30)	8 (89)	4 (44)
Anemia	39 (51)	4 (5)	16 (47)	3 (9)	17 (57)	0 (0)	5 (56)	1 (11)
Thrombocytopenia	52 (68)	19 (25)	21 (62)	8 (24)	22 (73)	6 (20)	8 (89)	5 (56)
**Non-hematological events**								
Nausea or vomiting	7 (9)	0 (0)	3 (9)	0 (0)	2 (7)	0 (0)	2 (22)	0 (0)
Alanine aminotransferase increased	20 (26)	2 (3)	9 (27)	1 (3)	7 (23)	1 (3)	3 (33)	0 (0)
Aspartate aminotransferase increased	18 (24)	1 (1)	8 (24)	1 (3)	6 (20)	0 (0)	3 (33)	0 (0)
γ-glutamyl transferase increased	7 (9)	0 (0)	4 (12)	0 (0)	2 (7)	0 (0)	1 (11)	0 (0)
Mucositis oral	3 (4)	0 (0)	1 (3)	0 (0)	0 (0)	0 (0)	2 (22)	0 (0)
Infection^b^	7 (9)	5 (7)	3 (9)	3 (9)	3 (10)	1 (3)	1 (11)	1 (11)
Cardiac toxicity	0 (0)	0 (0)	0 (0)	0 (0)	0 (0)	0 (0)	0 (0)	0 (0)
Neurological toxicity	4 (5)	0 (0)	2 (6)	0 (0)	1 (3)	0 (0)	1 (11)	0 (0)

Data are n (%)

*R-ICE* rituximab plus ifosfamide, carboplatin, and etoposide

^a^ Grading was performed according to the National Cancer Institute’s Common Terminology Criteria for Adverse Events (CTCAE v5.0). The CTCAE v5.0 grades the AEs based on their severity on a 5-point scale, with Grade≥3 indicating severe life-threatening and death

^b^ Includes catheter-related, lung, skin, upper respiratory tract, and urinary tract infections

### Mutation status

**Fig. 3:**
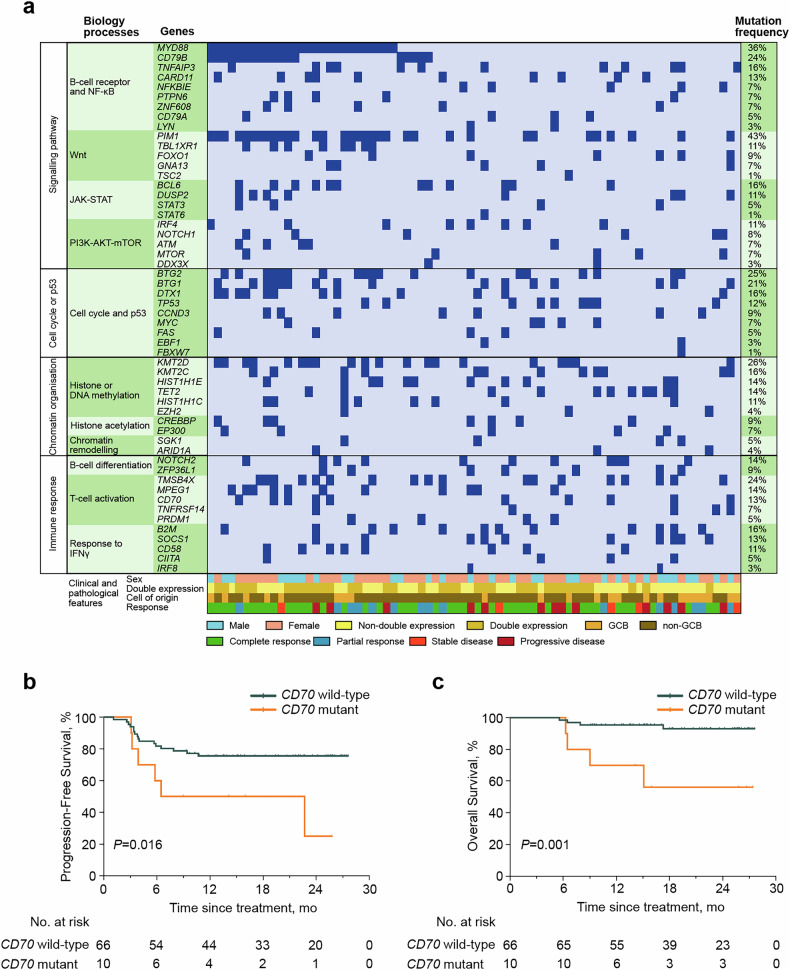
Mutation profile and clinical outcomes of patients with R/R DLBCL who received R-ICE-X. **a** Gene mutations identified by targeted sequencing in 76 patients. Genes mutated in at least one patient were shown. Mutation frequency is shown. **b** Progression-free survival of patients with *CD70* wild-type and *CD70* mutation. **c** Overall survival of patients with *CD70* wild-type and *CD70* mutation. *R/R* relapsed or refractory, *DLBCL* diffuse large B-cell lymphoma

Genomic analysis across the cohort (n = 76) identified pathogenic variants within DLBCL driver pathways, with mutations categorized by functional annotation in Fig. 3a and Supplementary Table 3. Mutation frequencies over 10% were observed for genes involved in the BCR and NF-κB pathway [*MYD88* (36%), *CD79B* (24%), *TNFAIP3* (16%), *CARD11* (13%)]; WNT pathway [*PIM1* (43%)*, TBL1XR1* (11%)]; JAK-STAT pathway [*BCL6* (16%)*, DUSP2* (11%)]; phosphatidylinositol 3 kinase-protein kinase B-mammalian target of rapamycin (PI3K-AKT-mTOR) pathway [*IRF4* (11%)]; cell cycle and p53 pathway [*BTG2* (25%)*, BTG1* (21%)*, DTX1* (16%)*, TP53* (12%)]; histone or DNA methylation [*KMT2D* (26%)*, KMT2C* (16%)*, HIST1H1E* (14%)*, TET2* (14%)*, HIST1H1C* (11%)]; B-cell differentiation [*NOTCH2* (14%)]; T-cell activation [*TMSB4X* (24%)*, MPEG1* (14%)*, CD70* (13%)]; and interferon (IFN)-γ response pathway [*B2M* (16%)*, SOCS1* (13%)*, CD58* (11%)]. Genes with recurrent mutations (≥10% prevalence) were analyzed for survival associations. Univariate Cox regression revealed *CD70* mutational status conferred significant progression-risk elevation (PFS: hazard ratio (HR) 3.00 [95% confidence interval (CI) 1.17–7.69], *P* = 0.022; OS: HR 8.06 [95% CI 2.00–32.52], *P* = 0.003). The 2-year PFS and OS rates were 25.0% (95% CI 1.6–62.9%) and 56.0% (95% CI 19.7–81.3%) in patients with *CD70* mutation, significantly lower than patients without *CD70* mutation (PFS, 75.6% [95% CI 63.3–84.3%], *P* = 0.016 and OS, 93.1% [95% CI 82.1–97.4%], *P* = 0.001) (Fig. 3b, c).

### Gene expression analysis

**Fig. 4:**
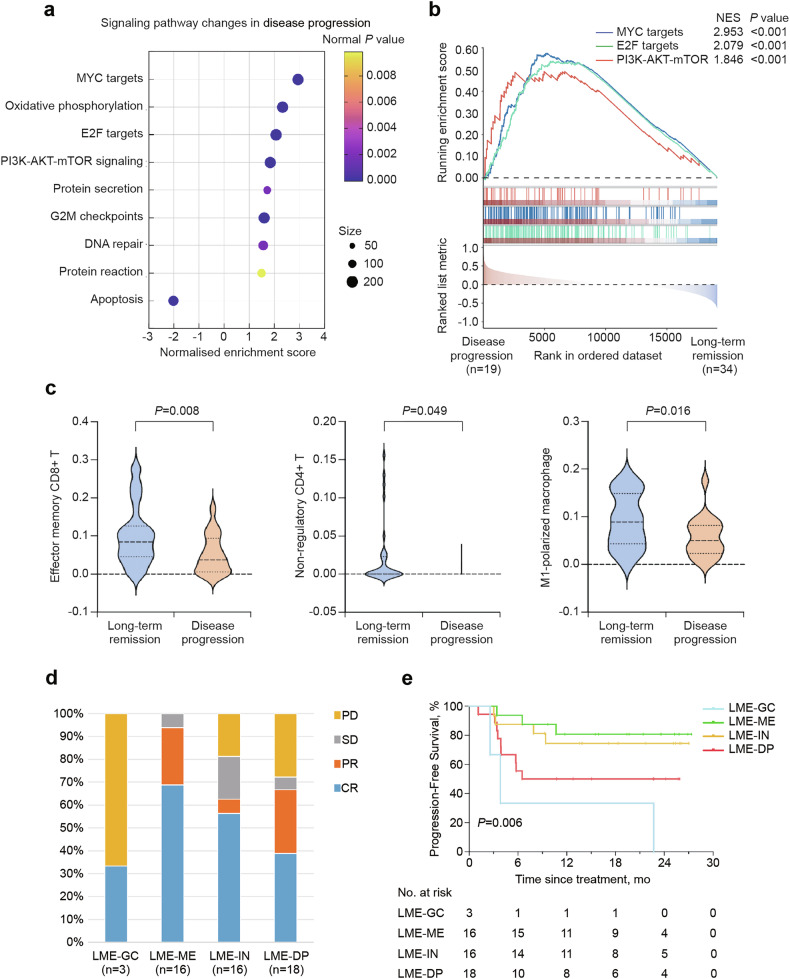
Potential resistance mechanism in disease progression to R-ICE-X. **a** Up-regulated GO pathways in patients with disease progression relative to those with long-term remission. **b** Comparison of gene set enrichment analysis in patients with disease progression relative to those with long-term remission. **c** Infiltration level of indicated immune cells in patients with disease progression relative to those with long-term remission. **d** Association of LME categories and related treatment response. **e** Kaplan-Meier analysis of progression-free survival according to LME categories. *R-ICE-X* rituximab, ifosfamide, carboplatin, and etoposide combined with targeted agent, *GO* Gene ontology, *NES* normalized enrichment score, *LME* lymphoma microenvironment

RNA sequencing data were available in 53 (70%) of 76 cases. Gene set enrichment analysis (GSEA) and gene ontology (GO) pathway analysis demonstrated significant activation of the PI3K-AKT-mTOR pathway (normalized enrichment score [NES] = 1.846, *P* < 0.001) in progressive disease subgroups. Additionally, several oncogenic transcription factors, including MYC (NES = 2.953, *P* < 0.001) and E2F (NES = 2.079, *P* < 0.001), known for promoting cell growth and proliferation, showed pronounced enrichment in progressive disease. In contrast, the apoptosis pathway was found to be enriched in patients achieving sustained remission (Fig. 4a, b).

### Lymphoma microenvironment

To characterize tumor immune microenvironmental heterogeneity, bulk transcriptomic deconvolution was performed using the xCell algorithm, providing comprehensive profiling of 64 immune-stromal cell subsets. Progressive disease was associated with reduced infiltration of CD8 + T cells (*P* = 0.008), CD4 + T cells (*P* = 0.049), and classically activated macrophages (M1 macrophages) cells (*P* = 0.016) (Fig. 4c). Low immune infiltrate density correlated with adverse outcomes, particularly CD8 + T cell depletion showing borderline significance (Supplementary Fig. 5). Meanwhile, using the lymphoma microenvironment (LME) categories, 3 (6%), 16 (30%), 16 (30%), and 18 (34%) patients were categorized into germinal center-like (LME-GC), mesenchymal (LME-MS), inflammatory (LME-IN), and depleted (LME-DP) respectively. LME-MS and LME-IN subtypes exhibited superior treatment response to R-ICE-X, with higher remission rates and prolonged PFS (*P* = 0.006, Fig. 4d, e).

## Discussion

R/R DLBCL patients exhibit dismal prognoses, with current salvage regimens demonstrating suboptimal clinical outcomes. To our knowledge, this study represents the first prospective investigation evaluating genotype-guided immunochemotherapy in R/R DLBCL management. The R-ICE regimen was incorporated into the National Comprehensive Cancer Network (NCCN) guidelines as the preferred second-line treatment. Historical data from the CORAL trial established reference outcomes for R/R DLBCL cohorts receiving R-ICE immunochemotherapy, demonstrating a CR rate of 24.4% and ORR of 63.5%.^6^ Considering the therapeutic stagnation in R/R DLBCL, this first phase II trial (NCT05348213) evaluated genotype-guided R-ICE-X immunochemotherapy. To prevent the delay of treatment caused by targeted sequencing analysis processes, we administered the R-ICE regimen for the first cycle to reduce the tumor burden and then used the simplified genetic algorithm LymphPlex to ensure the application of targeted agents combined with the R-ICE regimen for the second cycle. This approach achieved a median turnaround time of 14 days for actionable genomic reporting. Following the R-CHOP regimen, patients in ST2-like and EZB-like subtypes demonstrate superior survival than those in MCD-like, *TP53*^Mut^, and N1-like subtypes, whereas patients in the BN2-like subtype have great variation in survival outcomes across different cohorts.^9^ Therefore, MCD-like, BN2-like, and *TP53*^Mut^ emerged as the predominant genetic subtypes. The proportions of ST2-like and EZB-like subtypes were relatively low. Compared with previous salvage chemotherapy regimens (Supplementary Table 4), our study demonstrated that the R-ICE-X treatment (mainly R-ICE-zanubrutinib and R-ICE-lenalidomide regimen) significantly improved response rates, with an ORR of 76.3% (CR 56.6%) in both ASCT-eligible and ASCT-ineligible R/R DLBCL. Among 52 ASCT-eligible patients aged ≤65 years, 41/52 (79%) attained CR/PR, including refractory/relapsed disease within 12 months (40%) and relapsed disease more than 12 months (39%), further validating R-ICE-X as an effective bridging regimen to ASCT. Regarding the predictive factors, bulky disease is an unfavorable predictor of PFS and OS upon R-ICE-X treatment, highlighting the importance of integrating radiotherapy with chemotherapy.^17,18^ Additionally, bulky disease in lymphoma is associated with the high density of tumor-associated macrophages within the immunosuppressive tumor microenvironment, which contributes to the poor prognosis of the patients.^19^ The majority of AEs, primarily hematologic toxicities, were manageable and reversible with dose modifications and growth factor support, which is comparable to previously reported salvage regimens (Supplementary Table 4). Grade 3-4 non-hematological toxicities consisted primarily of hepatic transaminitis and infection, which were well-controlled, but still should be noted. No fatal serious AEs were reported in this study.

To investigate potential biomarkers of resistance, we evaluated the association between genomic alterations and disease progression. *CD70* mutations linked to poor prognosis in R/R DLBCL upon R-ICE-X treatment. CD70 demonstrates pathognomonic overexpression across diverse solid and hematologic neoplasms,^20^ inducing co-stimulation and promoting the differentiation of cytotoxic T lymphocyte cells. CD70 further promotes the activation of T cells by binding to its receptor CD27.^21^ When immune evasion results from *CD70* alterations, restoring T cell function may augment tumor immunity. Meanwhile, emerging translational validation from a preclinical study demonstrates that CD70-directed CAR-T cell constructs induce durable molecular remissions in CD19-negative DLBCL refractory to anti-CD19 cellular therapies, potentially through the reversal of antigen escape mechanisms via CD27 costimulatory signaling potentiation.^22^ In addition, up-regulated PI3K-AKT-mTOR pathways were observed in patients with disease progression. Persistent PI3K-AKT-mTOR pathway activation drives DLBCL progression through survival, proliferation, and metabolic reprogramming. Dysregulation stems from receptor mutations, phosphatase loss, and epigenetic alterations, sustaining kinase hyperactivity. This signaling cascade promotes therapeutic resistance via anti-apoptotic effects, unchecked cell division, and angiogenesis, contributing to R/R DLBCL aggressiveness.^23^ Clinically validated PI3K inhibitors and dual PI3K/mTOR antagonists show promising effects in molecularly defined subsets, bringing new treatment options for R/R DLBCL patients.^24^

The immunosuppressive tumor microenvironment facilitates immune evasion by suppressing cytotoxic T-cell activity and promotes tumor progression through impaired immune surveillance.^25^ In our cohort, patients exhibiting disease progression demonstrated markedly diminished infiltration of immune cell infiltration levers, such as CD8 + T, CD4 + T, and M1 subtype of macrophages, indicating the immunosuppressive tumor microenvironment precluded the effectiveness of R-ICE-X treatment. Zanubrutinib exerts multifaceted effects on both innate and adaptive immunity by inhibiting BCR signaling pathways while simultaneously reprogramming myeloid cell functionality.^26^ Lenalidomide potentiates T-cell-mediated antitumor responses through cereblon-dependent degradation of lymphoid transcription factors and enhancement of immune synapse formation.^27^ The combined treatment effects likely restore immune defenses by expanding tumor-infiltrating immune cell densities, explaining better treatment responses to the R-ICE-X regimen. Indeed, we discovered that patients with LME-ME and LME-IN subtypes benefited from R-ICE-X treatment (mainly treated with zanubrutinib and lenalidomide). Consistent with our previous findings, the LME-IN subtype associated with inflammatory cells, including CD8 + T cells, macrophages, and neutrophils, could be sensitive to zanubrutinib and lenalidomide treatment. Conversely, the LME-ME subtype has characteristics of high stromal cell infiltration and extracellular matrix pathway activation. Lenalidomide shows potential anti-fibrotic effects by inhibiting NF-κB signaling, and BTK inhibition significantly suppresses the attachment of migrating cells to extracellular matrix components or stromal cells.^28,29^ However, it should be mentioned that due to the favorable therapeutic effect of LME-GC upon the R-CHOP regimen, the proportion of LME-GC was relatively lower in our R/R cohort than in newly diagnosed DLBCL cohorts.^10,30^ All three patients with LME-GC were classified as MYC/BCL2 double expression, which potentially explained their aggressive clinical behavior and limited therapeutic response. Further studies are needed to elucidate in depth the impact of these targeted agents on the LME-GC subtype of R/R DLBCL.

This study had potential limitations. Our research, being a single-center study that provides hypothesis-generating insights, is inherently constrained by its non-randomized design and limited sample size. The results were interpreted in comparison with historical controls, which might potentially introduce a selection bias. Besides, adding new agents to conventional treatment could potentially interfere with the delivery of conventional treatment components. Given the small number of patients included in this study, further validation through prospective multicenter randomized controlled trials is necessitated. Nevertheless, these hypothesis-generating observations could serve as the foundation for developing rigorous protocols.

Collectively, this precision medicine paradigm validates R-ICE-X as a genotype-guided therapy, concurrently disrupting oncogenic signaling pathways and reprogramming immune-evasive niches, demonstrating clinically meaningful efficacy with a favorable safety profile in R/R DLBCL.

## Materials and Methods

### Study design and participants

This phase 2 single-arm, open-label trial (ClinicalTrials.gov NCT05348213) adopted a prospective cohort design. Enrollment was restricted to R/R DLBCL patients. Participant inclusion criteria included: age 18–75 years; had pathologically confirmed DLBCL, according to the World Health Organization (WHO) diagnostic criteria; an ECOG performance status 0–2; not achieving a CR/PR after the first line of chemoimmunotherapy or relapse/progression after remission; had an anticipated survival duration of longer than three months; and informed consent given. Participant exclusion criteria included: previous ASCT; central nervous system (CNS) involvement; had a left ventricular ejection fraction (LVEF) of 50% or less, as previously suggested;^31^ active systemic pathologies (poorly controlled cardiovascular/cerebrovascular disorders, refractory coagulopathies, autoimmune conditions requiring immunosuppression); chronic viral hepatitis with detectable HBV DNA; HIV seropositivity; gestational or lactational status and protocol nonadherence risks due to psychiatric comorbidities or undocumented contraindications. Eligibility criteria were detailed in the study protocol.

Written informed consent was obtained per the Declaration of Helsinki principles. Ethical approval was granted from Shanghai Ruijin Hospital’s Independent Ethics Committee prior to study initiation, with trial registration at ClinicalTrials.gov (NCT05348213).

### Randomization and masking

No blinding or randomization was performed since it was an open-label study. Therapeutic allocation awareness was maintained by both clinical investigators and enrolled participants.

### Procedures

Baseline diagnostic workup comprised standardized hematological profiling (complete blood count with differential), comprehensive metabolic panels including lactate dehydrogenase quantification, viral serology panels (HBV DNA quantification, HIV-related antibody), coagulation test, bone marrow biopsy and aspiration, cardiac functional imaging (electrocardiograph and echocardiography), and metabolic tumor volume assessment through fluorine-18 fluorodeoxyglucose positron emission tomography with computed tomography (18F-FDG PET/CT) or contrast-enhanced CT. IPI scores were conducted following the criteria described before.^32^

Two hematopathologists independently reviewed all histopathology data. GCB or non-GCB subtyping was determined by the Hans algorithm.^33^ In terms of double expressor, the cutoff value for BCL-2 was 50%, and MYC was 40% by immunohistochemistry.^34^ Dual-fusion/break-apart FISH probes were applied to detect BCL2, BCL6, and MYC translocations across the cohort. DNA sequencing variants were analyzed by the LymphPlex algorithm.^9^

The R-ICE immunochemotherapy protocol adhered to NCCN Guideline-specified dosing as follows: intravenous rituximab 375 mg/m² on day 0, intravenous ifosfamide 1500 mg/m² and etoposide 100 mg/m^2^ on day 1–3, intravenous carboplatin, area under curve (AUC)×[glomerular filtration rate (GFR, ml/min)+25] mg/d, AUC = 5, on day 2. New targeted agents were added based on genotyping results: zanubrutinib 160 mg orally twice a day on day 1-21 for MCD-like and BN2-like; lenalidomide 25 mg orally daily on day 1-10 for N1-like and NOS; decitabine 10 mg/m^2^ intravenously from day -5 to day -1 for *TP53*^Mut^; chidamide 20 mg orally daily on day 1, 4, 8, 11 for EZB-like; tofacitinib 5 mg orally twice a day daily on day 1-10 for ST2-like. The dosage of the investigational drug was designed based on previous literature reports.^10,35–38^ All patients received pegylated recombinant human granulocyte colony-stimulating factor (PEG-rhG-CSF) prophylaxis post-chemotherapy. The treatment was repeated every 21 days. Patients would undergo ultrasound assessments prior to the second cycle of chemotherapy, and contrasted CT after 2 treatment cycles, followed by a PET/CT evaluation after three treatment cycles for early detection of disease progression. Patients who achieved CR/PR after induction should consider ASCT for ASCT-eligible patients or continue three cycles of consolidation therapy with lenalidomide maintenance therapy for ASCT-ineligible counterparts. Patients who had SD or PD received other treatment regimens.

Grade 3-4 hematological or non-hematological toxicities mandated protocol-specified molecular therapeutic suspension until resolution to grade ≤1, with subsequent R-ICE cycle administration incorporating 20% dose attenuation. HBV carriers received entecavir prophylaxis with monthly viral load monitoring. Myeloid support via G-CSF was protocol-triggered when absolute neutrophil counts (ANC) sustained less than 1.0 × 10^9^ cells/L. Supportive treatment was at the discretion of the investigators. CNS prophylaxis incorporating high-dose methotrexate was given for patients with high-risk anatomical involvement (bone marrow infiltration, Waldeyer’s ring extension, orbital/paranasal sinus/testicular involvement). Involved-site radiotherapy (30–40 Gy) was delivered to patients with baseline metabolic tumor volume ≥7.5 cm or persistent metabolic activity on end-of-treatment 18F-FDG PET/CT.

### Endpoints and assessment

This investigation employed the primary endpoint of CR rate, assessed by PET/CT after three treatment cycles. The secondary endpoints comprised: the PFS rate, quantified from treatment initiation to radiographically confirmed progression or death from any cause; the OS rate, calculated from protocol enrollment to death; the ORR, incorporating participants with CR and PR per Deauville 5-point scale; and the toxicity, graded through National Cancer Institute Common Terminology Criteria of Adverse Events (NCI CTCAE) v5.0 evaluations at protocol-specified intervals. Exploratory biomarker analysis integrated DNA and RNA sequencing to detect resistance-associated genomic signatures.

PET/CT was assessed before treatment, at interim evaluations, and upon completion of therapy according to standardized criteria for non-Hodgkin lymphoma (Lugano classification).^39^ Contrast-enhanced CT encompassing cervical, thoracic, abdominal, and pelvic regions was repeated at protocol-defined intervals: every three months in the first year, every six months until two years, and every year thereafter to monitor disease progression.

### DNA sequencing

Genomic DNA isolation was performed using cryopreserved tumor specimens with the QIAamp DNA Mini Kit (Qiagen, Hilden, Germany) or formalin-fixed paraffin-embedded (FFPE) archival tissues processed through the GeneRead DNA FFPE Kit (Qiagen), employing manufacturer-prescribed enzymatic lysis and extraction guidelines under DNase/RNase-free conditions. Targeted sequencing (n = 76 FFPE samples) was conducted after DNA quality validation via agarose gel electrophoresis.^40^

### RNA sequencing

Stranded RNA sequencing libraries were constructed from cryopreserved tumor specimens (n = 53). Total RNA was isolated using Trizol, followed by RNeasy Mini Kit (Qiagen) purification, maintaining RNase-free conditions throughout. Sequencing was performed on an Illumina HiSeq 2000 platform, following RNA integrity verification (Agilent 2100 Bioanalyzer).^41^ Bioinformatic analyses were implemented in R v4.1.1, including batch effect correction through the “sva” package. Read counts were normalized and subjected to differential expression analysis using “limma” (v3.38.3). GSEA results were performed with statistical significance thresholds set at |log2(fold change)| ≥1 and Benjamini-Hochberg false discovery rate (FDR) < 0.05. Tumor heterogeneity was characterized using xCell (https://comphealth.ucsf.edu/app/xcell) to calculate cell type enrichment scores,^42^ and LME classification followed published methods. ^30^

### Genetic subtyping

Molecular stratification utilized the LymphPlex algorithm, which was a streamlined 38-gene panel detecting 35 somatic nonsynonymous variants through targeted sequencing and three translocations (*BCL2, BCL6*, and *MYC*) through FISH. The associated features for each category include: MCD-like (*BTG1, CD79B, IRF4, MYD88, MPEG1, PIM1, PRDM1, TBL1XR1* mutations), BN2-like (*BCL6* rearrangement, *BTG2, CD70, CCND3, DTX1, NOTCH2, TNFAIP3* mutations), N1-like (*NOTCH1* mutations), *TP53*^Mut^ (pathogenic *TP53* variants), EZB-like (*BCL2* rearrangement, *ARID1A, B2M, CIITA, CREBBP, EZH2, EP300, FAS, GNA13, KMT2D, STAT6, TNFRSF14* mutations, with or without *MYC* rearrangement), and ST2-like (*DDX3X, DUSP2, IRF8, SGK1, SOCS1, STAT3, TET2, ZFP36L1* mutations). Consistent with our previous results,^9^ algorithm validations against LymphGen classification demonstrated strong concordance. As quantitatively compared in Supplementary Fig. 6, the LymphPlex algorithm assigned subtypes in 66% (50/76) of cases, while the LymphGen algorithm assigned subtypes in 61% (46/76) of cases. Upon exclusion of *TP53*^Mut^ variants (LymphPlex) and A53 classifications (LymphGen), cross-algorithm concordance analysis demonstrated 97% subtype consistency across matched molecular categories: LymphPlex-defined MCD-like, BN2-like, N1-like, EZB-like, and ST2-like subtypes versus LymphGen-defined MCD, BN2, N1, EZB, and ST2 classifications. The only discordance involved one BN2-classified (LymphGen) case categorized as MCD-like by LymphPlex.

### Statistical analysis

A Simon’s two-stage optimal design was implemented, with prespecified efficacy thresholds (CR rate). Historical controls from R-ICE therapy in R/R DLBCL reported a CR/uncertain CR rate of 36%.^6^ We hypothesized a 20% absolute increase (H0: 36% vs. H1: 56%). Type I/II error probabilities were set at α = 0.05 (one-sided binomial test) and β = 0.20 (80% power). The first stage required enrollment of 23 evaluable patients, requiring ≥9 CR events to proceed to the second stage. Subsequent accrual permitted up to 68 additional patients. Accounting for a 10% anticipated dropout rate, the final sample size was inflated to 76 to power time-to-event analyses. Sample size estimation was performed using PASS software (version 11.0.10, NCSS, Kaysville, UT, USA).

Kaplan-Meier curves stratified by treatment subgroups estimated time-to-event outcomes. Survival distributions were assessed through the log-rank test. Prognostic effects were quantified using univariate or multivariable Cox proportional hazards regression, presenting HR with associated 95% CI. Nonparametric comparisons of immunophenotypic scores and log2-transformed gene expression utilized the Mann-Whitney U test. Statistical significance was defined as a two-tailed alpha threshold of 0.05. ITT analysis included all patients receiving at least one protocol cycle (n = 76 evaluable). The statistical computing was conducted using SPSS Statistics software (version 26.0) and GraphPad Prism (v10.1.1).

### Role of the funding source

The funders of the study were excluded from all methodological oversight, including trial design, data acquisition/curation, analytical frameworks, clinical interpretation, and manuscript development. All contributing authors maintained unrestricted data access privileges, exercised editorial autonomy over intellectual content, and retained ultimate accountability for publication decisions.

## Supplementary information

Supplementary Materials

Supplementary Table 1

Supplementary Table 3

Trial protocol

## Data Availability

Genomic and gene expression data have been deposited on GSA human (https://ngdc.cncb.ac.cn/gsa-human) under the project PRJCA041579. All data supporting the stated conclusions of the manuscript are in the paper or in the Supplementary Materials.
