# Supplementary material for: Genetic subtype-guided immunochemotherapy in relapsed and refractory diffuse large B cell lymphoma: a phase 2 investigator-initiated nonrandomized clinical trial (GUIDANCE-06)
Source: Signal Transduct Target Ther. 2025 Jul 26;10:232. doi: 10.1038/s41392-025-02316-6 (PMC12297320; doi:10.1038/s41392-025-02316-6)
Supplement: Supplementary file 1 — Supplementary Materials [file 41392_2025_2316_MOESM1_ESM.docx]

**Supplementary Materials for**

**Genetic subtype-guided immunochemotherapy in relapsed and refractory diffuse large B cell lymphoma: a phase 2 investigator-initiated nonrandomized clinical trial (GUIDANCE-06)**

Yi-Ge Shen ^1*^, Qing Shi ^1*^, Wei Tang ^1*^, Peng-Peng Xu ^1*^, Yi-Wen Cao ^1^, Meng-Meng Ji ^1^, Zhong Zheng ^1^, Shu Cheng ^1^, Li Wang ^1,2#^, Wei-Li Zhao ^1,2#^

**This PDF file includes:**

**Table S2, Tables S4**

**Figure S1 to Figure S6**

**Other Supplementary Materials for this manuscript include the following (separate file):**

Table S1. Clinical features of 76 patients with R/R DLBCL who received R-ICE-X.

Table S3. Genetic features and gene mutations of 76 patients with R/R DLBCL who received R-ICE-X.

| Category | Variables | Progression-free survival | |  | Overall survival | |
| --- | --- | --- | --- | --- | --- | --- |
|  |  | HR (95% CI) | P value |  | HR (95% CI) | P value |
| Age, y | ≤65y | 1 |  |  | 1 |  |
|  | >65y | 1.34 (0.56-3.19) | 0.51 |  | 0.70 (0.14-3.48) | 0.66 |
| Disease status | Refractory | 1 |  |  | 1 |  |
|  | Relapsed | 0.94 (0.41-2.17) | 0.88 |  | 1.56 (0.37-6.54) | 0.55 |
| Gender | Female | 1 |  |  | 1 |  |
|  | Male | 0.98 (0.42-2.30) | 0.97 |  | 0.85 (0.20-3.56) | 0.83 |
| ECOG performance status | 0-1 | 1 |  |  | 1 |  |
|  | 2 | 1.23 (0.36-4.16) | 0.74 |  | 2.65 (0.53-13.12) | 0.23 |
| Serum LDH | Normal | 1 |  |  | 1 |  |
|  | Elevated | 1.95 (0.58-6.58) | 0.28 |  | 2.08 (0.26-16.91) | 0.49 |
| Ann Arbor stage | I-II | 1 |  |  | 1 |  |
|  | III-IV | 1.02 (0.40-2.61) | 0.97 |  | 0.63 (0.15-2.64) | 0.53 |
| Extranodal sites | 0-1 site | 1 |  |  | 1 |  |
|  | ≥2 sites | 0.82 (0.35-1.92) | 0.64 |  | 1.34 (0.33-5.34) | 0.68 |
| Bulky disease | No | 1 |  |  | 1 |  |
|  | Yes | 2.89 (1.18-7.10) | 0.02 |  | 6.39 (1.59-25.63) | 0.01 |
| Systemic symptoms | Absence | 1 |  |  | 1 |  |
|  | Presence | 0.81 (0.34-1.89) | 0.62 |  | 0.99 (0.24-4.13) | 0.98 |
| IPI risk group | Low risk | 1 |  |  | 1 |  |
|  | Intermediate risk | 2.20 (0.50-9.74) | 0.30 |  | 1.19 (0.13-10.68) | 0.87 |
|  | High risk | 2.08 (0.43-10.03) | 0.36 |  | 1.73 (0.18-16.67) | 0.63 |
| Cell of origin | GCB | 1 |  |  | 1 |  |
|  | Non-GCB | 0.89 (0.36-2.18) | 0.80 |  | 1.21 (0.24-6.00) | 0.82 |
| BCL2/MYC DE | No | 1 |  |  | 1 |  |
|  | Yes | 1.64 (0.70-3.85) | 0.25 |  | 0.62 (0.15-2.59) | 0.51 |
| IHC MYC+ (>40%) | No | 1 |  |  | 1 |  |
|  | Yes | 1.26 (0.53-3.01) | 0.60 |  | 0.41 (0.10-1.72) | 0.22 |
| IHC BCL2+ (>50%) | No | 1 |  |  | 1 |  |
|  | Yes | 2.04 (0.60-6.88) | 0.25 |  | 1.96 (0.24-16.00) | 0.53 |
| ASCT | No | 1 |  |  | 1 |  |
|  | Yes | 0.20 (0.05-0.85) | 0.03 |  | 0.78 (0.16-3.85) | 0.76 |
| CAR-T | No | 1 |  |  | 1 |  |
|  | Yes | 6.84 (2.91-16.05) | <0.001 |  | 2.23 (0.53-9.35) | 0.27 |

**Table S2. Univariate analysis of prognostic factors in patients with R/R DLBCL who received R-ICE-X**

Abbreviations: HR, hazard ratio; CI, confidence interval; ECOG, eastern cooperative oncology group; IPI, International Prognostic Index; DE, double expression; LDH, lactate dehydrogenase; GCB, germinal center B-cell; IHC, immunohistochemistry; ASCT, autologous stem cell transplantation; CAR-T, chimeric antigen receptor T-cell immunotherapy.

| **Article** | Salvage regimens with autologous transplantation for relapsed large B-cell lymphoma in the rituximab era. | | Randomized Comparison of Gemcitabine, Dexamethasone, and Cisplatin Versus Dexamethasone, Cytarabine, and Cisplatin Chemotherapy Before Autologous Stem-Cell Transplantation for Relapsed and Refractory Aggressive Lymphomas: NCIC-CTG LY.12 | | Ofatumumab Versus Rituximab Salvage Chemoimmunotherapy in Relapsed or Refractory Diffuse Large B-Cell Lymphoma: The ORCHARRD Study. | |
| --- | --- | --- | --- | --- | --- | --- |
| **Author** | Gisselbrecht C, et al. | | Crump M, et al. | | Gustaaf W, et al. | |
| **ClinicalTrials.gov Identifier** | NCT 00137995/CORAL study | | NCT00078949/LY.12 study | | NCT01014208/ORCHARRD Study | |
| **Study phase** | Randomized Phase III | | Randomized Phase III | | Randomized Phase III | |
| **Patients** | 18-65 years, relapsed/refractory CD20+ B-cell lymphoma including DLBCL | | ≥18 years, relapsed/refractory aggressive NHL | | ≥18 years, relapsed/refractory CD20+ DLBCL or Grade 3B FL | |
| **Therapeutic regimen** | R-ICE | R-DHAP | (R)-GDP | (R)-DHAP | R-DHAP | O-DHAP |
| **Patient number** | 202 | 194 | 310 | 309 | 223 | 222 |
| **Median age (range)** | 54 (19-65) | 55 (19-65) | 55.2 (18.7-71.2) | 54.6 (22.6-74.3) | 56.0 (18-79) | 57.5 (23-83) |
| **DLBCL proportion** | / | / | 71.3% | 67.2% | 93.3% | 93.7% |
| **High-risk proportion** | 37.1% (saaIPI=2-3’) | 38.1% (saaIPI=2-3’) | 33.0% (IPI≥3’) | 32.5% (IPI≥3’) | 39.0% (saaIPI=2-3’) | 40.1% (saaIPI=2-3’) |
| **Dosage and administration** | Rituximab 375mg/m2 day0 Etoposide 100mg/m2/d days1-3  Ifosfamide 5000mg/m2 day2 Carboplatin AUC=5; maximum dose 800mg day2 | Rituximab 375mg/m2 day0 Cisplatin 100mg/m2 day1 Cytarabine 2g/m2 q12h day2 Dexamethasone 40mg/d days1-4 | Rituximab 375mg/m2 day0 (revised) Gemcitabine 1000mg/m2/d days1, 8 Cisplatin 75mg/m2 day1 Dexamethasone 40mg/d days1-4 | Rituximab 375mg/m2 day0 (revised) Cisplatin 100mg/m2 day1 Cytarabine 2g/m2 q12h day2 Dexamethasone 40mg/d days1-4 | Rituximab 375mg/m2 day0 Cisplatin 100mg/m2 day1 Cytarabine 2g/m2 q12h day2 Dexamethasone 40mg/d days1-4 | Ofatumumab 1000mg day0 Cisplatin 100mg/m2 day1 Cytarabine 2g/m2 q12h day2 Dexamethasone 40mg/d days1-4 |
| **Intervals and cycles** | 21 days; 3 cycles | 21 days; 3 cycles | 21 days; 2 cycles | 21 days; 2 cycles | 21 days; 3 cycles | 21 days; 3 cycles |
| **CRR (%)** | 48/197 (24.4%) | 53/191 (27.7%) | 41/303 (13.9%) | 44/302 (14.6%) | 48/223 (21.5%) | 34/222 (15.3%) |
| **ORR (%)** | 125/197 (63.5%) | 120/191 (62.8%) | 140/303 (46.2%) | 135/302 (44.7%) | 94/223 (42.2%) | 84/222 (37.8%) |
| **PFS** | 3y-PFS: 31% | 3y-PFS: 42% | HR=0.99; 95%CI=0.82-1.21; P=0.95 | | 2y-PFS: 26% | 2y-PFS: 24% |
| **OS** | 3y-OS: 47% | 3y-OS: 51% | HR=1.03; 95%CI=0.83-1.28; P=0.78 | | 2y-OS: 38% | 2y-OS: 41% |
| **Transplant proportion** | 51.3% | 55.0% | 52.1% | 49.3% | 36.8% | 33.3% |
| **PFS of transplant patient** | 3y-PFS: 53% | / | / | | 2y-PFS: 52% | 2y-PFS: 50% |
| **OS of transplant patient** | / | / | / | | 2y-OS: 68% | 2y-OS: 76% |
| **≥ Grade 3 hematological toxicity** |  | |  | |  | |
| **Neutropenia** | 23% (grade 3-4) | 24% (grade 3-4) | 15% (grade 3-4) | 32% (grade 3-4) | 26% (grade 4) | 22% (grade 4) |
| **Thrombocytopenia** | 35% required platelet transfusion | 57% required platelet transfusion | / | / | 35% (grade 4) | 35% (grade 4) |
| **Anemia** | / | / | / | / | / | / |
| **≥ Grade 3 non-hematological toxicity** |  | |  | |  | |
| **Infection related** | 17% (grade 3-4) | 16% (grade 3-4) | 13% (grade 3-4) | 16% (grade 3-4) | 13% (SAEs) | |
| **Neurological toxicity** | / | / | / | / | / | / |
| **Cardiac toxicity** | / | / | 2% (grade 3-4) | 5% (grade 3-4) | / | / |
| **Renal insufficiency** | 1% (grade 3-4) | 6% (grade 3-4) | / | / | 5% (SAEs) | |
| **Elevation of aminotransferase** | / | / | / | / | 16% (any grade) | 23% (any grade) |
| **Nausea or vomiting** | / | / | 11% (grade 3-4) | 15% (grade 3-4) | 5% (SAEs) | |
| **Conclusion** | No difference was observed between the effects of R-ICE and R-DHAP. | | Treatment with GDP before HDT-ASCT is as effective as DHAP and associated with fewer adverse events, less frequent need for hospitalization, and superior patient-reported QoL. | | O-DHAP and R-DHAP as salvage treatment was not able to overcome treatment resistance of relapsed or refractory DLBCL. | |
|  |  |  |  |  |  |  |

**Table S4. Published clinical trials on second-line treatment options in patients with R/R DLBCL**

**
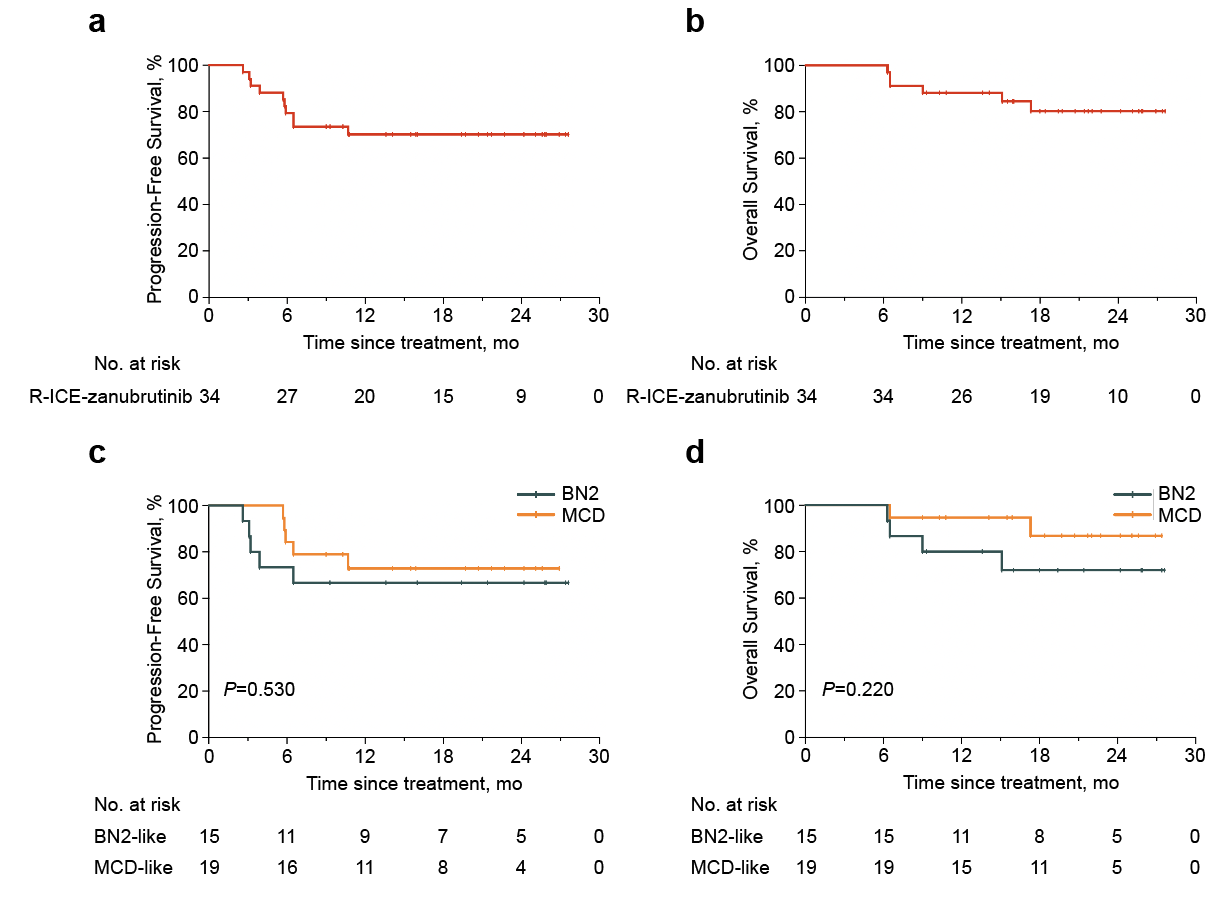
**

**Figure S1. Progression-free survival and overall survival of patients with R/R DLBCL who received R-ICE-zanubrutinib**

(a) Progression-free survival of patients receiving R-ICE-zanubrutinib. (b) Overall survival of patients receiving R-ICE-zanubrutinib. (c) Progression-free survival of patients in BN2-like and MCD-like subtypes. (d) Overall survival of patients in BN2-like and MCD-like subtypes.

**
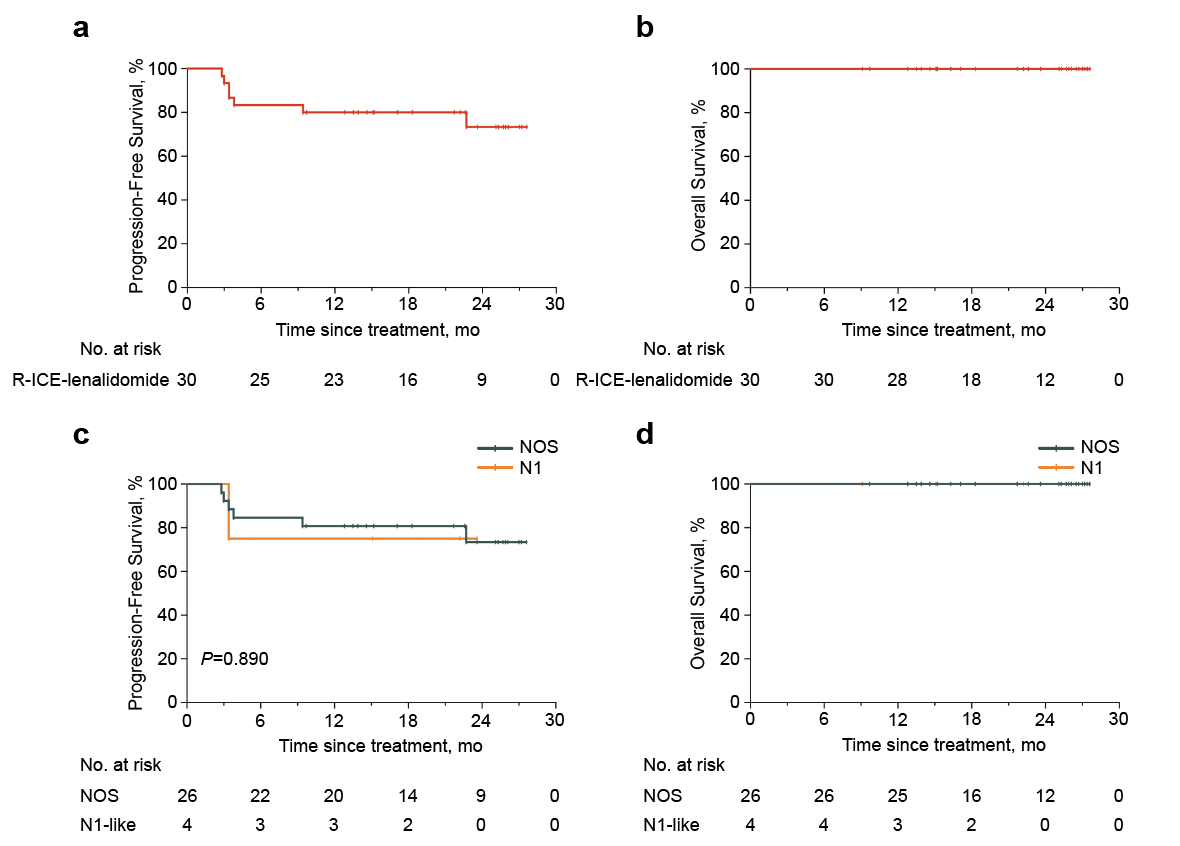
**

**Figure S2. Progression-free survival and overall survival of patients with R/R DLBCL who received R-ICE-lenalidomide**

(a) Progression-free survival of patients receiving R-ICE-lenalidomide. (b) Overall survival of patients receiving R-ICE-lenalidomide. (c) Progression-free survival of patients in NOS and N1-like subtypes. (d) Overall survival of patients in NOS and N1-like subtypes.

**
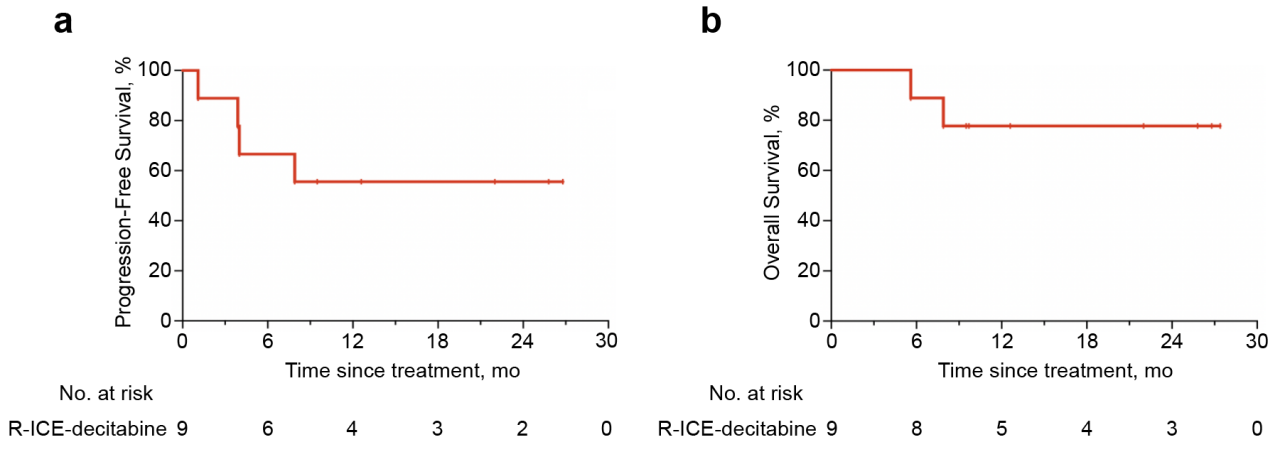
**

**Figure S3. Progression-free survival and overall survival of patients with R/R DLBCL who received R-ICE-decitabine**

(a) Progression-free survival of patients receiving R-ICE-decitabine. (b) Overall survival of patients receiving R-ICE-decitabine.


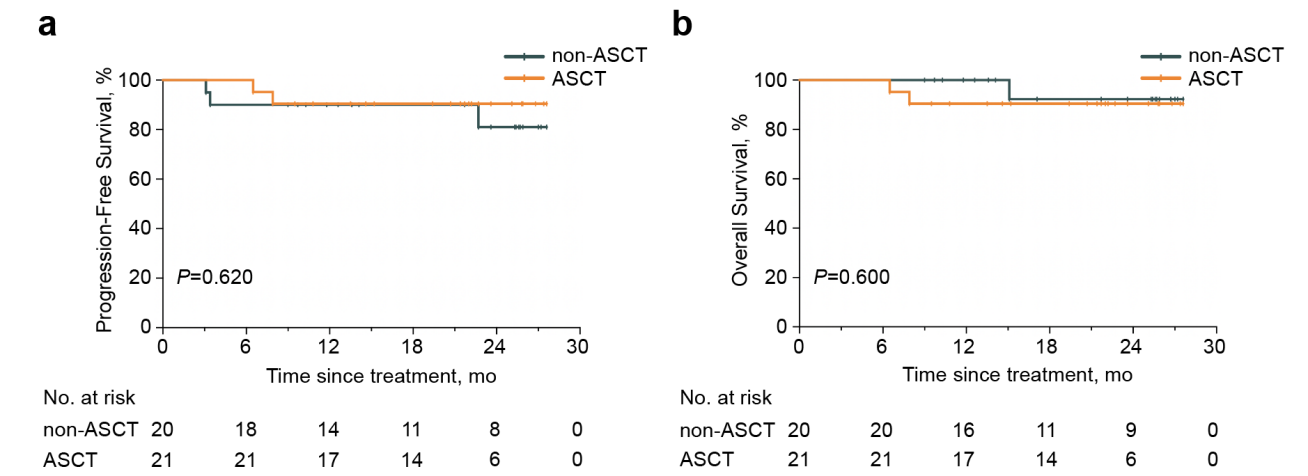


**Figure S4. Comparison of progression-free survival and overall survival in patients of ASCT group and non-ASCT group**

1. Progression-free survival of patients proceeding to ASCT and patients without ASCT. (b) Overall survival of patients proceeding to ASCT and patients without ASCT.

Abbreviation: ASCT, autologous stem cell transplantation.


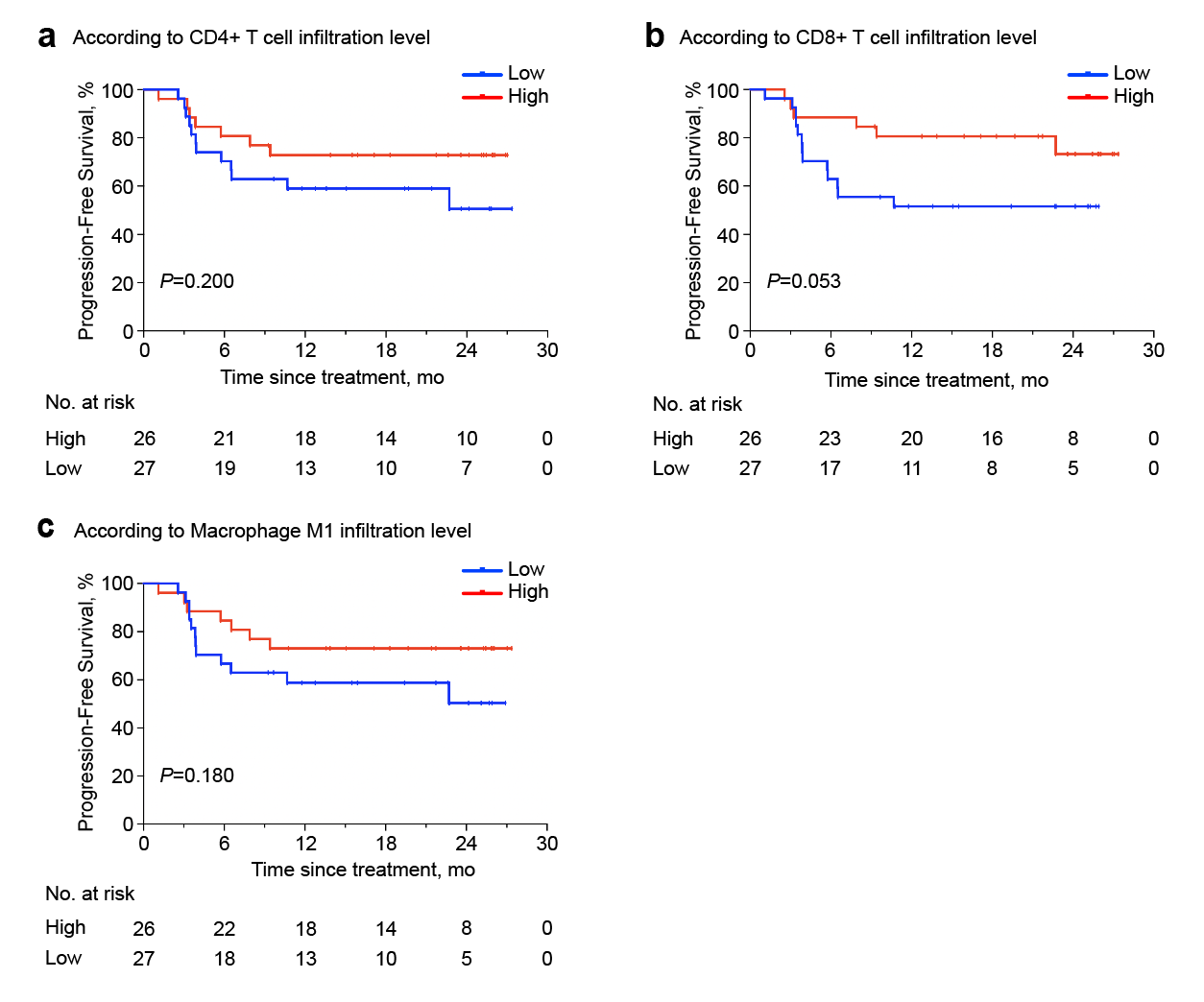


**Figure S5. Survival analysis according to immune cell infiltration in patients with R/R DLBCL who received R-ICE-X**

(a-c) Kaplan-Meier plots showing progression-free survival for patients stratified by the level of CD8+ T, CD4+ T, and M1 macrophages cell infiltration.


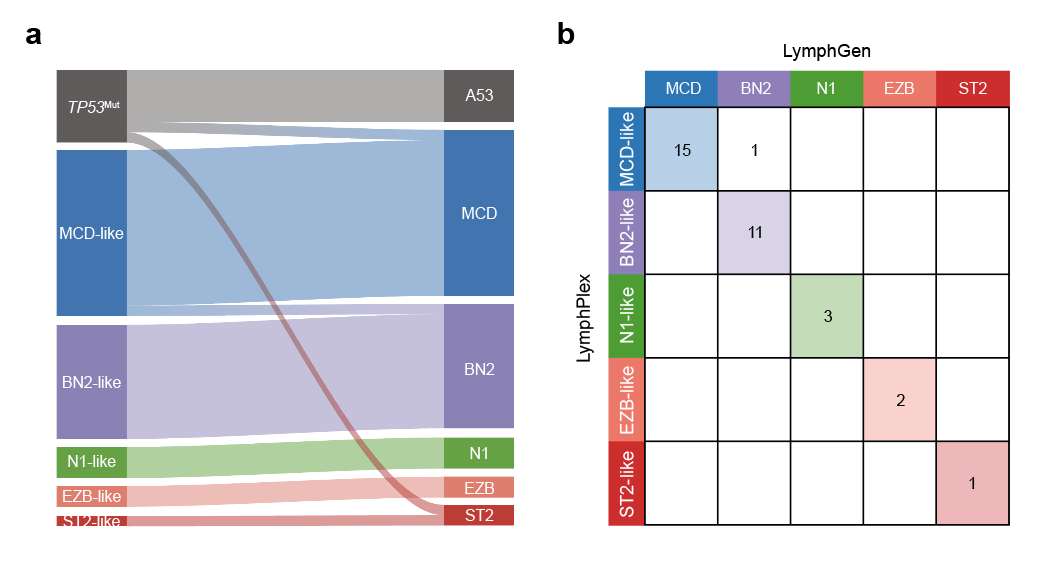


**Figure S6. Validation of the genetic subtyping using LymphPlex algorithm and LymphGen algorithm**

1. Sankey plots showing the results of the LymphPlex algorithm and the LymphGen algorithm in patients for whom a unique genetic subtype was assigned by both algorithms. (b) Confusion matrixes showing numbers of patients for each subtype assigned by the LymphPlex algorithm and the LymphGen algorithm.
