# Supplementary material for: Genetic subtype-guided immunochemotherapy in relapsed and refractory diffuse large B cell lymphoma: a phase 2 investigator-initiated nonrandomized clinical trial (GUIDANCE-06)
Source: Signal Transduct Target Ther. 2025 Jul 26;10:232. doi: 10.1038/s41392-025-02316-6 (PMC12297320; doi:10.1038/s41392-025-02316-6)
Supplement: Supplementary file 4 — Trial protocol [file 41392_2025_2316_MOESM4_ESM.docx]

**Genetic subtype-guided immunochemotherapy in relapsed and refractory diffuse large B cell lymphoma: a Phase 2 investigator-initiated nonrandomized clinical trial (GUIDANCE-06)**

CONFIDENTIAL

The information contained in this document is regarded as confidential and, except to the extent necessary to obtain informed consent, may not be disclosed to another party unless law or regulations require such disclosure. Persons to whom the information is disclosed must be informed that the information is confidential and may not be further disclosed by them.

Protocol No. R-ICE-X

Investigator's Brochure

Protocol Version 1.1 – Dated Jan 4, 2022

**SCHEMA**

**PROTOCOL TITLE:** Clinical Study of Efficacy and Safety of Novel Targeted Drugs Combined With R-ICE Regimen in the Treatment of Relapsed and Refractory Diffuse Large B-cell Lymphoma

**PROTOCOL NUMBER:** version 1.1

**STUDY DRUG:** Zanubrutinib, Lenalidomide, Decitabine, Chidamide, Tofacitinib

R-ICE: Rituximab, Ifosfamide, Carboplatin, and Etoposide

**INDICATION:** Diffuse large B-cell lymphoma

**STUDY PHASE:** Ⅱ

**BACKGROUND AND RATIONALE:**

Diffuse large B cell lymphoma (DLBCL) is one of the most common types of aggressive B-cell non-Hodgkin lymphoma (NHL). It is a general term for a group of highly heterogeneous malignant clonal diseases, accounting for 31% to 35% of NHL. Rituximab combined with CHOP chemotherapy (rituximab, cyclophosphamide, vincristine, doxorubicin, and prednisone) in the treatment of DLBCL has a cure rate of about 60%, and 5-year PFS has increased from 37% in 1975 to 66% in 2005. The advent of RCHOP has improved the survival of DLBCL patients, but 30%-40% of patients still respond poorly to treatment and relapse or die in the short term.

Various chemotherapy regimens such as ICE, DHAP, ESHAP, and GDP have been tried to be used in second-line treatment, and there is no significant difference in efficacy and toxicity of these regimens. Among them, etoposide, isocyclophosphamide, and carboplatin (ICE) schemes are more commonly used, with ORR of about 50-70% and CR of 25-30% in relapsed/refractory (R/R) DLBCL patients. Therefore, there is a substantial unmet need for exploring novel effective and safe treatment options regimens for R/R DLBCL patients.

In recent years, with the development of high-throughput sequencing technology, people's understanding of the molecular variation map of DLBCL has gradually deepened, and targeted drugs targeting related pathogenic pathways have been developed and marketed successively. The clinical treatment status and prognosis of different pathogenic subtypes of DLBCL are gradually different. In 2020, Cancer Cell published an article integrating previous research results, which divided DLBCL into seven subtypes: MCD, N1, A53, BN2, ST2, EZB (MYC+), and EZB (MYC-). It also further revealed the targeted drugs that could potentially benefit different subtypes of DLBCL in the era of "R-CHOP+X". This has given a basis for encouraging targeted therapies added to second-line therapy.

We intend to design this prospective, single-arm, single-center exploratory clinical trial to determine the efficacy and safety of novel targeted drugs combined with R-ICE (R-ICE-X) in R/R DLBCL patients with different molecular subtypes, providing a new approach to improve the prognosis and survival of DLBCL patients.

**STUDY OBJECTIVES:**

**Primary:**

(1) To assess the complete response rate (CRR) of novel targeted drugs combined with R-ICE (R-ICE-X) in R/R DLBCL patients.

**Secondary:**

(1) To assess the overall response rate (ORR) of the R-ICE-X regimen in R/R DLBCL patients.

(2) To assess the progression-free survival (PFS) in patients with R/R DLBCL receiving the R-ICE-X regimen.

(3) To assess the overall survival (OS) in patients with R/R DLBCL receiving R-ICE-X regimen.

(4) To evaluate the number of participants with treatment-related adverse events (AEs) as assessed by Common Terminology Criteria Adverse Events (CTCAE) Version 5.0.

**STUDY DESIGN:**

This is a prospective, single-arm, open label, phase Ⅱ study of novel targeted drugs combined with R-ICE (R-ICE-X) regimen, aiming to evaluate the efficacy and safety of this treatment therapy in relapsed/refractory diffuse large B-cell lymphoma (DLBCL) patients. Approximately 76 patients will be enrolled. The eligible patients are to receive R-ICE-X based on genotyping results every 21 days for 3 cycles. Interim evaluation is performed after 3 cycles. If the interim evaluation indicates complete or partial response (CR/PR), the patients will consider autogenetic stem-cell transplantation (ASCT) or continue 3 cycles of consolidation therapy with lenalidomide maintenance therapy. Patients who had stable or progressive disease (SD/PD) received other treatment regimens.

**STUDY ENDPOINTS:**

**Primary:**

The primary endpoint for the study will be the complete response rate.

**Secondary:**

Secondary endpoints will include:

1. Overall response rate

2. Progression-free survival

3. Overall survival

4. Number of Participants with Treatment-Related Adverse Events as Assessed by CTCAE v5.0

**Exploratory:**

The exploratory Endpoint for the study will be the evaluation of potential predictive biomarkers

for drug response or resistance.

**STUDY DURATION:** Expected to be completed within 2 years for enrollment, and follow-up will be conducted for 2 years after enrollment.

**TOTAL SAMPLE SIZE:** The trial will include approximately 76 patients.

**DOSING REGIMEN(S):**

1. Rituximab 375 mg/m² administered intravenously each 21-day treatment cycle on day 0
2. Ifosfamide 1500 mg/m² and etoposide 100 mg/m^2^ intravenously on days 1-3
3. Carboplatin AUC×[GFR(ml/min)+25] mg/d, AUC=5, intravenously on day 2
4. New targeted drugs were added based on genotyping results:

Zanubrutinib 160mg orally twice daily on days 1-21 for MCD-like and BN2-like

Lenalidomide 25mg orally daily on day 1-10 for N1-like and NOS

Decitabine 10mg/m^2^ intravenously from day -5 to day -1 for *TP53*^Mut^

Chidamide 20mg orally daily on days 1,4,8,11 for EZB-like

Tofacitinib 5mg orally twice a day daily on days 1-10 for ST2-like

**TABLE OF CONTENTS**

SCHEMA ..................................................................................................................................................2

1. OBJECTIVES .......................................................................................................................................6

1.1 Primary Objectives...............................................................................................................................6

1.2 Secondary Objectives ..........................................................................................................................6

1.3 Exploratory Objectives .......................................................................................................................6

1.4 Primary Endpoint ................................................................................................................................6

1.5 Secondary Endpoints ...........................................................................................................................6

1.6 Exploratory Endpoint ..........................................................................................................................6

2. BACKGROUND ..................................................................................................................................7

2.1 Study Population .................................................................................................................................7

2.2 Second-line treatment regimen ...........................................................................................................7

2.3 Targeted therapy .................................................................................................................................7

2.4 Investigational Agent ..........................................................................................................................8

2.5 Research Purpose ................................................................................................................................8

3. PATIENT SELECTION........................................................................................................................9

3.1 Inclusion Criteria ................................................................................................................................9

3.2 Exclusion Criteria ...............................................................................................................................9

4. TREATMENT PLAN .........................................................................................................................10

4.1 Overall Design ..................................................................................................................................10

4.2 Dosing regimen .................................................................................................................................10

4.3 Dose reduction protocols for cytotoxic drugs ...................................................................................10

4.4 Treatment cycles ...............................................................................................................................11

4.5 Visit schedule and assessments .........................................................................................................11

4.6 General Concomitant Medication and Supportive Care Guidelines .................................................13

4.7 Discontinuation of Study Treatment .................................................................................................13

5. EFFICACY EVALUATION ..............................................................................................................14

5.1 Evaluation criteria .............................................................................................................................14

5.2 Evaluation methods ...........................................................................................................................14

6. ADVERSE EVENTS REPORTING REQUIREMENTS ...................................................................15

6.1 Definition ..........................................................................................................................................15

6.2 Attribution of the AE ........................................................................................................................15

6.3 Serious AE ........................................................................................................................................15

6.4 Reporting process ..............................................................................................................................16

7. STUDY CALENDAR .........................................................................................................................17

7.1 Observation items and time points ....................................................................................................17

7.2 Study process ....................................................................................................................................18

8. DATA REPORTING / REGULATORY CONSIDERATIONS ........................................................19

8.1 Trial quality control and assurance ...................................................................................................19

8.2 Data Collection .................................................................................................................................19

9. STATISTICAL CONSIDERATIONS ................................................................................................20

9.1 General considerations ......................................................................................................................20

9.2 Datasets to be analyzed .....................................................................................................................20

9.3 Study endpoints .................................................................................................................................20

9.4 Statistical methods ............................................................................................................................20

9.5 Sample size considerations ...............................................................................................................20

REFERENCES .......................................................................................................................................22

APPENDICES ........................................................................................................................................23

APPENDIX 1. Evaluation Criteria for Malignant Lymphoma (Lugano Criteria) ..................................23

APPENDIX 2. NHL efficacy criteria (excluding PET-CT) ...................................................................25

APPENDIX 3. NHL efficacy criteria (including PET-CT) ....................................................................26

**1 OBJECTIVES**

**1.1 Primary Objectives**

(1) To assess the complete response rate (CRR) of novel targeted drugs combined with R-ICE (R-ICE-X) in R/R DLBCL patients.

**1.2 Secondary Objectives**

(1) To assess the overall response rate (ORR) of R-ICE-X regimen in R/R DLBCL patients.

(2) To assess the progression-free survival (PFS) in patients with R/R DLBCL receiving the R-ICE-X regimen.

(3) To assess the overall survival (OS) in patients with R/R DLBCL receiving the R-ICE-X regimen.

(4) To evaluate the number of participants with treatment-related adverse events (AEs) as assessed by Common Terminology Criteria Adverse Events (CTCAE) Version 5.0.

**1.3 Exploratory Objectives**

(1) To evaluate the mechanism of the R-ICE regimen and the potential predictive biomarkers for drug response or resistance, including DNA and RNA sequencing.

**1.4 Primary Endpoint**

(1) Complete response rate

**1.5 Secondary Endpoints**

(1) Overall response rate

(2) Progression-free survival

(3) Overall survival

(4) Number of participants with treatment-related adverse events as assessed by CTCAE v5.0

**1.6 Exploratory Endpoint**

(1) Evaluation of potential predictive biomarkers for drug response or resistance

**2. BACKGROUND**

**2.1 Study Population**

Diffuse large B cell lymphoma (DLBCL) is one of the most common types of aggressive B-cell non-Hodgkin lymphoma (NHL). It is a general term for a group of highly heterogeneous malignant clonal diseases, accounting for 31% to 35% of NHL.^1^ A retrospective analysis of big data in the past 10 years in China found that DLBCL accounted for 57% of B-cell lymphoma, higher than the incidence of 30% in European and American countries. Rituximab combined with CHOP chemotherapy (rituximab, cyclophosphamide, vincristine, doxorubicin, and prednisone) in the treatment of DLBCL has a cure rate of about 60%, and 5-year PFS has increased from 37% in 1975 to 66% in 2005. This result led to R-CHOP becoming the standard first-line treatment for DLBCL.^2,3^ The advent of RCHOP has improved the survival of DLBCL patients, but 30%-40% of patients still respond poorly to treatment and relapse or die in the short term.^4^

**2.2 Second-line treatment regimen**

Various chemotherapy regimens such as ICE, DHAP, ESHAP, and GDP have been tried to be used in second-line treatment, and there is no significant difference in efficacy and toxicity of these regimens. Among them, etoposide, isocyclophosphamide, and carboplatin (ICE) schemes are more commonly used, with ORR of about 50-70% and CR of 25-30% in relapsed/refractory (R/R) DLBCL patients.^5^ A total of 400 patients with R/R DLBCL enrolled in Coral study were randomly divided into R-DHAP group and R-ICE group. After three courses of chemotherapy, the CR rate in the R-ICE group was 24% and CRu was 12%.^6^ Therefore, there is a substantial unmet need for exploring novel effective and safe treatment options regimens for R/R DLBCL patients.

**2.3 Targeted therapy**

In recent years, with the development of high-throughput sequencing technology, people's understanding of the molecular variation map of DLBCL has gradually deepened, and targeted drugs targeting related pathogenic pathways have been developed and marketed successively. The clinical treatment status and prognosis of different pathogenic subtypes of DLBCL are gradually different. In 2020, Cancer Cell published an article integrating previous research results, which divided DLBCL into seven subtypes: MCD, N1, A53, BN2, ST2, EZB (MYC+), and EZB (MYC-). It also further revealed the targeted drugs that could potentially benefit different subtypes of DLBCL in the era of "R-CHOP+X".^7^ This has given a basis for encouraging targeted therapies added to second-line therapy.

**2.4 Investigational Agent**

***Zanubrutinib***

Zanubrutinib is a selective inhibitor of Bruton's tyrosine kinase (BTK). It inhibits tyrosine phosphorylation at site 223 by covalently binding to cystine at site 481 of BTK protein, thereby inhibiting BTK activity.^8^ BTK is a key regulator of B cell receptor (BCR) signaling pathway and plays an important role in the proliferation, apoptosis, differentiation, and development of B cells.^9^ In vivo studies showed that zanubrutinib inhibited the growth of Rec-1 suite cell lymphoma cells and TMD-8 diffuse large B lymphoma cells in a dose-dependent manner.^8^ Zanubrutinib has been approved for the treatment of mantle cell lymphoma and chronic lymphocytic leukemia/small lymphocytic lymphoma (CLL/SLL).

***Lenalidomide***

Immunomodulatory drugs lenalidomide and pomalidomide are synthetic compounds derived by modifying the chemical structure of thalidomide to improve its potency and reduce its side effects. It is approved by FDA for clinical use in myelodysplastic syndromes with deletion of chromosome 5q and multiple myeloma. Lenalidomide is an immunomodulator, affecting both cellular and humoral limbs of the immune system. It has also been shown to have anti-angiogenic properties. Lenalidomide inhibits TNF-α production, stimulates T cells, reduces serum levels of the cytokines vascular endothelial growth factor (VEGF) and basic fibroblast growth factor (bFGF), and inhibits angiogenesis.^10^

***Decitabine***

Decitabine is a synthetic pyrimidine analogue with strong antitumor activity. It is a potent DNA methylation inhibitor, which can inhibit the growth and proliferation of tumor cells by interfering with the DNA methylation process and affecting gene expression.^11^ Studies have found that low dose decitabine can enhance the chemosensitivity of various cancer cells. Several clinical trials have confirmed that decitabine has a significant therapeutic effect on a variety of tumors, such as acute myeloid leukemia (AML), myelodysplastic syndrome (MDS), chronic myeloid leukemia (CML), etc. In addition, DNA methyltransferase inhibitors could affect R/R DLBCL growth and overcome chemotherapy resistance.

***Chidamide***

Chidamide is a novel orally active benzamide-type histone deacetylase inhibitor that has shown in vitro activities against a wide array of neoplasms. By inhibiting related HDAC subtypes, chidamide triggers chromatin remodeling, and thus produces changes in gene expression targeting multiple signal transmission pathways, thus inhibiting the tumor cell cycle and inducing tumor cell apoptosis. Meanwhile, chidamide could induce and enhance the tumor-killing mediated by natural killer cells (NK) and antigen-specific cytotoxic T cells (CTL).^12^ Chidamide is the first oral subtype-selective histone deacetylase inhibitor (HDACi) approved in China as well as the first HDACi of the benzamide class approved for the treatment of relapsed and refractory peripheral T-cell lymphoma (PTCL).^13^

***Tofacitinib***

Tofacitinib is an oral JAK inhibitor indicated for the treatment of rheumatoid arthritis (RA). JAK is a class of kinases involved in regulating the growth and differentiation of immune cells. By blocking the activity of JAK, tofacitinib reduces the production of inflammatory factors and regulates the immune system. In vitro study suggests that tofacitinib may represent a useful therapeutic agent for patients with EBV-associated T and NK cell lymphoma.^14^

**2.5 Research Purpose**

We intend to design this prospective, single-arm, single-center exploratory clinical trial to determine the efficacy and safety of novel targeted drugs combined with R-ICE (R-ICE-X) in R/R DLBCL patients with different molecular subtypes, providing a new approach to improve the prognosis and survival of DLBCL patients.

**3. PATIENT SELECTION**

**3.1 Inclusion Criteria**

Patients must satisfy all the following criteria to be enrolled in the study:

(1) According to the World Health Organization (WHO) classification of diseases, histologically confirmed DLBCL (except primary central lymphoma and HIV-related lymphoma)

(2) Evaluable lesions were detected by PET/CT

(3) Life expectancy of at least 3 months

(4) Previous first-line anti-lymphoma therapy and adequate treatment, no remission or disease progression, and no current anti-lymphoma therapy (≥2 weeks since the last anti-lymphoma therapy). Patients were allowed to receive hormones or rituximab at least 1 week of enrollment for symptom control reasons;

(5) Age 18-75 years old (including 18 and 75 years old)

(6) ECOG performance status 0, 1, 2

(7) Participate in the clinical trial voluntarily and sign an informed consent form

**3.2 Exclusion Criteria**

(1) Lymphoma patients with central nervous system (CNS) invasion

(2) Known allergy to test drug ingredients

(3) Uncontrollable cardio-cerebral vascular, coagulative, autoimmune, or serious infectious disease

(4) Laboratory measures meet the following criteria at screening (unless caused by lymphoma):

Neutrophile <1.5×10^9^/L

Platelet <75×10^9^ /L

Alanine aminotransferase (ALT) or aspartate aminotransferase (AST) >2×upper limit of normal (ULN)

Alkline phosphatase (AKP) or bilirubin >2×ULN

Creatinine clearance>60ml/min

(5) HIV infection

(6) If HBsAg positive, should check hepatitis B virus (HBV) DNA, DNA positive patients cannot be enrolled. If HBsAg negative but HBcAb positive (whatever HBsAb status), should check HBV DNA, DNA positive patients cannot be enrolled

(7) Pregnant or lactating women

(8) Other uncontrollable medical conditions that may interfere with the participation of the study

**4. TREATMENT PLAN**

**4.1 Overall design**

Refractory recurrence of patients with diffuse large B cell lymphoma inclusion/exclusion standard subjects in a signed written informed consent, according to the results of genotyping, divided the patients into MCD-like, *TP53*^Mut^, BN2-like, EZB-like, ST2-like, NOS, N1-like, a total of 7 kinds of types. New targeted drugs were added based on typing results: Zanubrutinib for MCD-like and BN2-like, Decitabine for *TP53*^Mut^, Chidamide for EZB-like, Tofacitinib for ST2-like, and Lenalidomide for N1-like and NOS. Patients would undergo ultrasound assessments prior to the second cycle of chemotherapy, and contrasted CT after 2 treatment cycles. After 3 courses of treatment, PET-CT was used to evaluate the efficacy. Patients with CR/PR, aged ≤65 years, qualified and willing for transplantation, were treated with autologous hematopoietic stem cell transplantation (ASCT); Patients who did not meet the above requirements or failed to collect autologous hematopoietic stem cells were placed on continuing 3 cycles of consolidation therapy and lenalidomide maintenance therapy used for a maximum of 12 months; If patients were evaluated as SD and PD after 3 cycles, they were dropped out of the trial and treated with other regiments. New targeted drugs combined with R-ICE and ASCT were evaluated every 3 months in the first year and every 6 months in the second and third years after treatment.

**4.2 Dosing regimen**

| **Drug name** | **Dosage** | **Route** | **Time of administration** |
| --- | --- | --- | --- |
| Rituximab | 375 mg/m^2^ | ivgtt. | Day 0 |
| Ifosfamide | 1500 mg/m^2^ per day | ivgtt. | Day 1-3 |
| Etoposide | 100 mg/m^2^ per day | ivgtt. | Day 1-3 |
| Carboplatin | AUC×[GFR(ml/min)+25]mg/d, AUC=5 | ivgtt. | Day 2 |
| The new targeted drug dosage is as follows: | | | |
| Zanubrutinib | 160 mg twice a day | p.o. | Day 1-21 |
| Lenalidomide | 25 mg per day | p.o. | Day 1-10 |
| Decitabine | 10 mg/m^2^ per day | ivgtt. | Day-5-Day-1 |
| Chidamide | 20 mg per day | p.o. | Day 1,4,8,11 |
| Tofacitinib | 5 mg twice a day | p.o. | Day 1-10 |

**4.3 Dose reduction protocols for cytotoxic drugs**

For the occurrence of adverse reactions in the course of treatment, the following principles of drug regulation are adopted:

In principle, it is not allowed to change the treatment plan for grade 1-2 AE related to therapeutic drugs. Whether symptomatic treatment is necessary is decided by the researcher according to the clinical situation. ≥Grade 3 therapeutically-associated hematologic AE and non-hematologic AE, adequate symptomatic supportive treatment is required, accompanied by the suspension of novel targeted drugs and/or dose reduction of subsequent R-ICE cycle.

| AE | Novel targeted drugs | R-ICE regimen |
| --- | --- | --- |
| Grade 3 | The medication was suspended and resumed when AE≤grade 2, and the original dose was maintained | Reduce the dose level in subsequent cycles.  (Dose reduction by 20%) |
| Grade 4 | The medication was suspended and resumed when AE≤grade 2, and the original dose was maintained | Reduce the dose level in subsequent cycles. (Dose reduction by 20%) |

**4.4 Treatment cycles**

R/R DLBCL patients who met eligibility criteria and signed informed consent received R-ICE-X treatment for 3 cycles per 21 days. According to the genetic subtyping results, new targeted drugs were added: Zanubrutinib for MCD-like and BN2-like subtypes, Decitabine for *TP53*^Mut^ subtype, Chidamide for EZB-like subtype, Tofacitinib for ST2-like subtype and Lenalidomide for N1-like and NOS subtypes. Patients who achieved CR or PR after 3 cycles of immunochemotherapy, continue to undergo ASCT or 3 more cycles of immunochemotherapy. The subjects will continue to receive regular evaluations after completion of treatment until the specified follow-up period ends (total study duration is 2 years).

**4.5 Visit schedule and assessments**

Screening Assessments and all on-study scheduled visits and assessments are outlined in the SECTION 7 STUDY CALENDAR.

**Baseline Evaluations**

All baseline evaluations are to be done within 4 weeks of the start of therapy, unless otherwise specified.

- All patients must have biopsy slides available demonstrating diffuse large B-cell lymphoma with confirmation of CD20 expression and other characteristic immunophenotyping

- Complete blood count (CBC) with differential and platelets

- Serum chemistries to include LDH

- Physical examination with history, vital signs, height, weight and performance status

- FDG-PET/CT extending from skull base to mid-thigh

- Bone marrow aspirate and core biopsy (within 8 weeks of treatment)

- History taking (including concurrent diseases and treatment)

- ECOG

- CRP

- NT pro-BNP

- DIC (including APTT, PT, and fibrinogen)

- Viral panel: HBV marker, HBV-DNA, HCV and HIV antibody

- Urine and stool analysis

- ECG

- UCG

- GI endoscopy (if necessary)

**Evaluations During Treatment**

On Day 1 of each cycle:

- Physical examination with vital signs and performance status

- ECOG

- Adverse events

- Serum chemistries to include LDH

- Urine and stool analysis

- Coagulation function

- CRP

- NT pro-BNP

- ECG

- UCG

- Concurrent diseases and treatment

- Viral panel: HBV marker, HBV-DNA, EBV-DNA, HCV, HIV

**Prior to cycle 4**

- FDG-PET/CT scan

- Bone marrow aspirate and core biopsy (if necessary)

- MR and ultrasound (if necessary)

- GI endoscopy (if necessary)

**Induction Completion**

After 6 cycles of treatment or discontinuation of treatment, patients will have the following evaluations:

- Physical examination with vital signs and performance status

- ECOG

- Serum chemistries to include LDH

- Urine and stool analysis

- CRP

- NT pro-BNP

- FDG-PET/CT scan

- If previously positive, bone marrow aspiration and core biopsy (unilateral) only if otherwise in

complete remission (not repeated once negative)

- Concurrent diseases and treatment

- Adverse events

**Follow Up**

Patients will have follow-up evaluations every 3 months post induction to monitor disease

progression until 1 year, then every 6 months until 2 years, and every year thereafter.

At each follow-up evaluation, the patient will have the following:

- Physical examination with vital signs and performance status

- ECOG

- Serum chemistries to include LDH

- Urine and stool analysis

- Coagulation function

- CRP

- NT pro-BNP

- ECG

- UCG

- HBV-DNA

- Concurrent diseases and treatment

- Contrast-enhanced CT scan of neck, thorax, abdomen, and pelvis (and other areas if indicated to evaluate sites of disease involvement)

- Adverse events

**4.6 General Concomitant Medication and Supportive Care Guidelines**

For patients with high tumor burden (bulky disease or LDH≥500 IU/L), ECOG performance status≥2, or gastrointestinal lymphoma (to prevent GI perforation), allopurinol and sodium bicarbonate should be given. Prednisone is taken orally to prevent tumor lysis syndrome, and chemotherapy will be reduced or divided into 2 days if necessary.

Granulocyte colony-stimulating factor (G-CSF): Long-acting G-CSF therapy will be given if necessary, according to investigators' discretion. The application of G-CSF should be recorded in the Conditional Random Field (CRF) table. No prophylactic use of G-CSF is suggested in this clinical trial.

Patients with positive serology of hepatitis B virus should be given entecavir or other similar antivirals to prevent hepatitis reactivation.

Prophylaxis for CNS relapse will be given to patients with involvement of bone marrow, nasal or paranasal sinuses, orbit, breast, kidney, adrenal gland, or testis.

For patients with large masses, it is up to the investigator to decide whether radiotherapy is needed.

Concomitant therapy given as a result of an adverse event should also be reported if it meets the reporting criteria, which should be provided on the AEs page of the CRF form. If necessary, patients can be given adequate supportive care, including transfusion of whole blood and blood products, antibiotic therapy, and antiemetic therapy. The reason for treatment, dosage, and the date of treatment should also be recorded in the CRF form.

**4.7 Discontinuation of Study Treatment**

Treatment will continue until patients have completed therapy or the occurrence of any of the following events:

-Disease progression or start of any other anti-lymphoma treatment

-Adverse event(s) that, in the judgment of the investigator, may cause severe or permanent harm or which rules out the continuation of the treatment regimen

-Discontinuation of any investigational drugs, such as unacceptable side events

-Major violation of the study protocol

-Withdrawal of consent

-Lost to follow-up

-Death

**5. EFFICACY EVALUATION**

A routine evaluation is performed before each cycle of immunochemotherapy, and an objective and comprehensive evaluation of efficacy is performed after 3 cycles of immunochemotherapy and before ASCT. Follow-up is conducted every 3 months until 2 years, and a second summary assessment is performed.

**5.1 Evaluation criteria**

PET-CT will be performed according to the revised criteria for the evaluation of the efficacy of malignant lymphoma in 2007. The assessment of response includes CR, PR, stable (SD), disease recurrence (post-CR), or disease progression (post-PR, post-SD). See Appendix 1-3 for details. The response of those who underwent enhanced CT scans will be evaluated according to the criteria for the efficacy assessment criteria for adult indolent and progressive NHL established by U.S. and international lymphoma experts in 1999; The assessment of response includes CR, unconfirmed complete response (CRu), PR, SD, post-CR, or post-PR, post-SD.

**5.2 Evaluation methods**

Evaluation of the tumor before and after treatment, including physical examination, CT/MRI, and PET-CT is recommended.

**6. ADVERSE EVENT REPORTING REQUIREMENTS**

**6.1 Definition**

An adverse event (AE) is any undesired medical event related to or unrelated to treatment that occurs in the clinical trial after treatment. It can be any adverse and unexpected signs, symptoms, laboratory examinations, and other abnormal results, whether or not they are treatment-related. The severity of AEs is graded according to the grading method recommended by the NCI CTC (5th Edition) Common Toxicity Criteria Grading system. All AEs should be recorded in the CRF table. AEs (such as blindness, neurotoxicity, loss of limbs, etc.) that are not resolved during the clinically possible observation period are classified as permanently unresolved, and the time for resolution of the event is left blank in the CRF table.

**6.2 Attribution of the AE**

CTCAE term (AE description) and grade: The descriptions and grading scales found in the revised NCI Common Terminology Criteria for Adverse Events (CTCAE) version 5.0 will be utilized for AE reporting. A copy of the CTCAE version 5.0 can be downloaded from the CTEP website (http://ctep.cancer.gov). The investigator should evaluate the possible association between AEs and the investigational drug and the combination drug with reference to the following 5-level classification criteria:

Definite related-The AE is clearly related to the study treatment. The reaction occurs in accordance with the reasonable time sequence after administration, and the reaction is consistent with the known safety events of the study drug. The reaction is improved after drug withdrawal and reappears after repeated administration.

Probable related-The AE is likely related to the study treatment. The reaction occurs in accordance with the reasonable time sequence after administration, and the reaction is consistent with the known safety events of the study drug. The patient's clinical status or other treatment modalities may also cause this event.

Possible unrelated-The AE may not be related to the study treatment. Reactions appear to be less consistent with a reasonable time sequence after administration, and reactions are less consistent with the known type of reaction of the study drug. The patient's clinical status or other treatment modalities may also cause this event.

Unrelated-The AE is unrelated to the study treatment. The reactions do not conform to the reasonable time sequence after administration, and the reactions are consistent with the known reaction types of other drugs. The patient's clinical status or other treatment modalities may produce the response, the disease status improves or discontinuation of the drug (other than the study drug) results in improvement of safety events, and the safety events reappear with the reuse of the suspected drug.

Unknown-The occurrence of the reaction is not clearly related to the time sequence after the administration of the study drug, and is similar to the type of reaction known for the study drug, and other drugs used at the same time may cause the same reaction.

Items 1), 2) and 5) above are recorded as treatment-related AEs in this study. Incidence of adverse reactions=Number of adverse reactions/total number of patients×100%.

**6.3 Serious AE**

A serious adverse event (SAE) is defined as an undesired AE that occurs at any drug dose and meets one of the following criteria:

1. Results in death
2. Life-threatening caused by the experimental drug
3. Lead to hospitalization or prolonged hospitalization
4. Permanent or significant loss of function/disability

5) Teratogenic or carcinogenic

Some events that require hospitalization or prolonged hospitalization may not be reported as serious AEs, including:

1. Hospitalization for social reasons other than AEs
2. Hospitalization for elective surgery, tests, or other treatments scheduled before entering the study

3) Progression of lymphoma and related events

**6.4 Reporting process**

**6.4.1 All AEs**

All AEs during the trial should be reported. Serious adverse events are reported using the SAE Report form. Toxicity will be scored using CTCAE Version 5.0 for toxicity and adverse event reporting. A copy of the CTCAE Version 5.0 can be downloaded from the CTEP homepage (http://ctep.info.nih.gov). All adverse clinical experiences, whether observed by the investigator or reported by the patient, must be recorded, with details about the duration and intensity of each episode, the action taken with respect to the test drug, and the patient’s outcome. The investigator must evaluate each adverse experience for its relationship to the test drug and its seriousness. The investigator must appraise all abnormal laboratory results for their clinical significance. If any abnormal laboratory result is considered clinically significant, the investigator must provide details about the action taken with respect to the test drug and the patient’s outcome.

**6.4.2 SAEs**

When a SAE occurs (drug-related or unrelated), the investigator should report it within 24 hours. Researchers should record the occurrence time, duration, actions taken, and outcome of serious adverse events in the report, signed and dated. Disease progression (including signs and symptoms of progression) is not reported as SAE, but deaths due to disease progression during the trial period should be reported as SAE. The Sponsor will ensure that all legal and regulatory requirements for reporting procedures are met.

**7. RESEARCH CALENDAR**

**7.1 Observation items and time points**

| **Evaluations** | **Screening** | **Induction treatment** | | | | **consolidation** | **ASCT** | **Maintenance**  **and follow up** |
| --- | --- | --- | --- | --- | --- | --- | --- | --- |
| Written  informed  consent |  | C1  D1 | C2  D1 | C3  D1 | End-of  induction  treatment | C4-C6 |  |  |
| History  taking | × |  |  |  |  |  |  |  |
| Bone  marrow  aspiration  and biopsy^a^ | × |  |  |  |  |  | × |  |
| Tissue  biopsy | × |  |  |  |  |  |  |  |
| Physical  examination | × | × | × | × | × | × | × | × |
| ECOG | × | × | × | × | × | × | × | × |
| Routine test  of blood,  urine and  feces | × | × | × | × | × | × | × | × |
| Coagulation  function | × | × | × | × | × | × | × | × |
| Biochemical  tests^b^ | × | × | × | × | × | × | × | × |
| CRP | × | × | × | × |  | × | × | × |
| NT pro  BNP | × | × | × | × |  | × | × | × |
| ECG | × | × | × | × |  | × | × | × |
| Echocardio-  graphy | × | × | × | × |  | × | × | × |
| HBV, HCV,  HIV  serologies | × | × | × | × |  | × | × | × |
| HBV DNA | × | × | × | × |  | × | × | × |
| Whole-body  PET/CT | × |  |  |  | × |  |  |  |
| Neck,  thoracic,  abdominal  and pelvic  CT scan  with  contrast^c^ |  |  | × |  |  |  | × | × |
| Concurrent  diseases and  treatment | × | × | × | × | × | × | × | × |
| Adverse  events |  | × | × | × | × | × | × | × |

a: within 8 weeks of starting treatment

b: including pre-albumin, Albumin, alkaline phosphatase, total bilirubin, bicarbonate, BUN, calcium, chloride, creatinine, glucose, LDH, phosphorus, potassium, total protein, AST, ALT, sodium, uric acid, serum B2-MG

c: every 3 months thereafter until 1 year, then every 6 months until 2 years, and every year thereafter

**7.2 Study process**


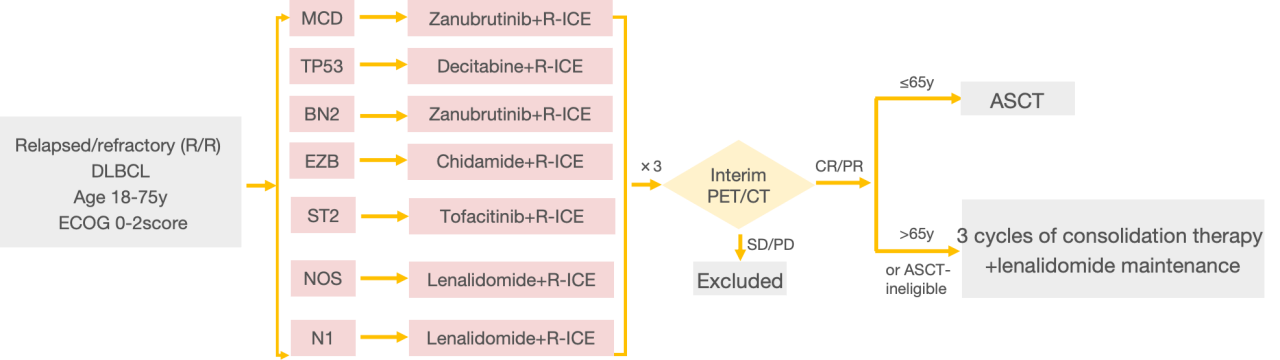


**8. DATA REPORTING / REGULATORY CONSIDERATIONS**

**8.1 Trial quality control and assurance**

In order to ensure that this study is carried out in strict accordance with the clinical trial protocol, clinical investigators, sponsors, and clinical monitors should operate in strict accordance with the requirements of GCP during the whole process of this study. It is necessary to ensure that the test procedures are standardized, the test data are accurate and the study conclusions are reliable. All medical records can be confirmed as valid only after the final data is verified and qualified.

**8.2 Data Collection**

***Analyses and Reporting***

The final analysis will be realized when the last patient finishes induction treatment. The approximate schedule of the final analysis will be approximately 30 months after the first enrollment.

***Data Monitoring Committee***

Data regarding safety will be reviewed at a weekly research meeting, where all patients in the study are reviewed and discussed. Any unexpected safety issues or issues with protocol compliance or deviation will be identified at this meeting. The data collection plan for this study is to utilize the online database to capture all treatment, toxicity, and efficacy data for all enrolled patients.

**9. STATISTICAL CONSIDERATIONS**

**9.1 General considerations**

Statistical analysis will be conducted by or under the supervision of the study statistician. Statistical analysis will be performed using SPSS.

**9.2 Datasets to be analyzed**

Up to 76 evaluable patients will be enrolled. All enrolled patients will follow the protocol with available primary endpoint data that can be used for efficacy analysis. All enrolled patients who receive at least one cycle of the R-ICE-X regimen are considered evaluable for safety analysis. Additional subjects will be enrolled to replace any subjects who are enrolled but do not receive their assigned treatment.

**9.3 Study endpoints**

The primary endpoint will be the complete response rate (CRR). Secondary endpoints will include overall response rate (ORR), progression-free survival (PFS), overall survival (OS), and adverse events.

**9.3.1 Primary endpoint**

**Complete response rate (CRR)** will be calculated as the proportion of participants with complete response at the end of R-ICE-X induction determined on the basis of investigator assessments according to the 2014 Lugano criteria.

**9.3.2 Secondary endpoints**

**1) Progression-free survival (PFS)** is defined as the time from diagnosis to the first observation of documented disease progression/relapse or death due to any cause. If a subject has not progressed or died, PFS will be censored at the time of the last visit with adequate assessment.

**2) Overall survival (OS)** will be measured from the date of diagnosis to the date of death from any cause. Alive patients will be censored at their last contact.

**3) Overall response rate (ORR)** will be calculated as the proportion of patients having a response (complete response or partial response) at the end of R-ICE-X induction.

**4) The number of participants with treatment-related adverse events** will be assessed by CTCAE v5.0.

**9.4 Statistical methods**

Descriptive statistics will be used to summarize the characteristics of the patients. Survival estimates will be calculated with the use of the Kaplan-Meier method and survival curves will be compared by the log-rank test. Hazard ratios and associated 95% confidence intervals will be estimated with the use of a univariate Cox proportional hazards model. A two-sided P value of < 0.05 is considered significant.

**9.5 Sample size considerations**

Previous studies showed that the CR rate of R-ICE for patients with relapsed/refractory DLBCL was approximately 36%, and we conservatively assumed the complete response rate was 56%. The probabilities of a one-sided Type I error (falsely accepting a non-promising therapy) and Type II error (falsely rejecting a promising therapy) were set to 0.05 and 0.2, respectively. In the first stage of this design, 23 patients were to be treated. If nine or more patients had a response, then up to 68 additional patients were to be accrued in the second stage. A drop-out rate of 10% was assumed, and therefore it was calculated that about 76 patients were needed. The sample size was calculated by PASS software (version 11.0.10, NCSS, Kaysville, UT, USA).

**REFERENCES**

1. Susanibar-Adaniya, S. & Barta, S. K. 2021 Update on Diffuse large B cell lymphoma: A review of current data and potential applications on risk stratification and management. *Am J Hematol*. **96**, 617-629 (2021).
2. Pfreundschuh, M. et al. CHOP-like chemotherapy plus rituximab versus CHOP-like chemotherapy alone in young patients with good-prognosis diffuse large-B-cell lymphoma: a randomised controlled trial by the MabThera International Trial (MInT) Group. *Lancet Oncol*. **7**, 379-391 (2006).
3. Hawkes, E. et al. AvR-CHOP: Feasibility Study of Induction and Maintenance Avelumab Plus R-CHOP in Patients with Diffuse Large B-Cell Lymphoma (DLBCL). *Blood* **134**, 5332-5332 (2019).
4. Coiffier, B. et al. Long-term outcome of patients in the LNH-98.5 trial, the first randomized study comparing rituximab-CHOP to standard CHOP chemotherapy in DLBCL patients: a study by the Groupe d'Etudes des Lymphomes de l'Adulte. *Blood* **116**, 2040-2045 (2010).
5. Moskowitz, C. H. et al. Ifosfamide, carboplatin, and etoposide: a highly effective cytoreduction and peripheral-blood progenitor-cell mobilization regimen for transplant-eligible patients with non-Hodgkin's lymphoma. *J Clin Oncol*. **17**, 3776-3785 (1999).
6. Gisselbrecht, C. et al. Salvage regimens with autologous transplantation for relapsed large B-cell lymphoma in the rituximab era. *J Clin Oncol.* **28**, 4184-4190 (2010).
7. Wright, G. W. et al. A Probabilistic Classification Tool for Genetic Subtypes of Diffuse Large B Cell Lymphoma with Therapeutic Implications. *Cancer Cell* **37**, 551-568.e14 (2020).
8. Guo, Y. et al. Discovery of Zanubrutinib (BGB-3111), a Novel, Potent, and Selective Covalent Inhibitor of Bruton's Tyrosine Kinase. *J Med Chem*. **62**, 7923-7940 (2019).
9. Hendriks, R. W., Yuvaraj, S. & Kil, L. P. Targeting Bruton's tyrosine kinase in B cell malignancies. *Nat Rev Cancer.* **14**, 219-232 (2014).
10. Venugopal, S., Mascarenhas, J. & Steensma, D. P. Loss of 5q in myeloid malignancies-A gain in understanding of biological and clinical consequences. *Blood Adv*. **46**, 100735 (2021).
11. Jabbour, E., Issa, J. P., Garcia-Manero, G. & Kantarjian, H. Evolution of decitabine development: accomplishments, ongoing investigations, and future strategies. *Cancer* **112**, 2341-2351 (2008).
12. Guan, X., Hua-Qing, W., Jia, L. & Liu, F. The Novel HDAC Inhibitor Chidamide Synergizes with Rituximab to Inhibit DLBCL Tumor Growth in Vitro and In Vivo By up-Regulating CD20 Expression. *Blood* **132** (Supple 1), 2947 (2018).
13. Hu, M., et al. A Single-Center Retrospective Clinical Study of Chidamide in the Treatment of Adult Peripheral T-Cell Lymphoma. *Blood* **138** (Supple 1), 4551 (2021).
14. Ando, S. et al. Tofacitinib induces G1 cell-cycle arrest and inhibits tumor growth in Epstein-Barr virus-associated T and natural killer cell lymphoma cells. *Oncotarget*. **7**, 76793-76805 (2016).

**Appendix 1: Evaluation Criteria for Malignant Lymphoma (Lugano Criteria)**

| Response | Position | PET-CT (metabolic reaction) | CT (morphological response) |
| --- | --- | --- | --- |
| Complete remission | Extranodal position and nodules | 5-PS score 1-3, regardless of whether there is a lump residue | (1) LDi of target lesion (lymph node mass) was reduced to less than 1.5 cm; (2) No extralymphatic lesions |
|  | Unmeasured lesion | - | None |
|  | Organ enlargement | - | Reduce to normal |
|  | New lesion | None | None |
|  | Bone marrow | No evidence of FDG uptake | Normal morphology (If the morphology is uncertain, flow cytometry is negative.) |
| Partial remission | Extranodal position and nodules | 5-PS score of 4-5, and decreased uptake compared with baseline; (2) No new or progressive lesions; (3) These findings suggest that treatment is effective at the interim assessment and that residual disease remains at the end-of-treatment assessment. | All conditions were met: (1) SPD decreased by more than 50% in at least 6 measurable lesions; (2) If the lesion is too small to be measured by CT, it should be treated by 5mm×5mm; (3) When not visible, according to 0mm×0mm processing; (4) When the lesion is larger than 5mm×5mm, but smaller than the normal value, the actual measured value is treated |
|  | Unmeasured lesion | - | None or normal or reduced, no increase |
|  | Organ enlargement | - | The portion of the spleen that exceeds its normal size has shrunk by at least 50%. |
|  | New lesion | None | None |
|  | Bone marrow | Uptake is higher than normal bone marrow but lower than baseline, and diffuse uptake may be increased due to reactive hyperplasia after chemotherapy; If focal uptake changes persist even after treatment for lymphadenopathy, further biopsy or periodic scanning may be considered. | - |
| Stable disease | Target lymph node (lymph node mass), extranodal lesion | 5-PS score of 4-5, no significant difference from baseline; (2) No new or progressive lesions. | Up to 6 measurable major lymph nodes or extranodal lesions had SPD reductions of less than 50% from baseline; (2) Did not meet the PD standard. |
|  | Unmeasured lesion | - | Does not meet PD standards |
|  | Organ enlargement | - | Does not meet PD standards |
|  | New lesion | None | None |
|  | Bone marrow | No change from baseline | - |
| Progressive disease | Target lymph node (lymph node mass), extranodal lesion | (1) A 5-PS score of 4-5, a significant increase from baseline uptake, and/or (2) Mid-term or post-treatment examination reveals new FDG uptake lesions consistent with lymphoma changes. | At least 1: (1) PPD progress; (2) Individual lymph nodes/lesions abnormal; (3) LDi >1.5cm and PPD increased 50% from the lowest point and LDi or SDi increased 0.5cm(for <2cm lesions) or 1cm(for >2cm lesions) from the lowest point; (4) Splenomegaly: If the original spleen is large, the degree of splenomegaly exceeds the original 50%. If there is no splenomegaly, the splenomegaly is at least 2cm above baseline. (5) New or re-emerging splenomegaly. |
|  | Unmeasured lesion | None | New, unmeasured lesions or significant progression of preexisting lesions |
|  | New lesion | (1) New FDG uptake lesions that cannot be explained by other causes (such as infection, inflammation, etc.); (2) If the cause cannot be determined, biopsy or regular follow-up scans should be considered. | (1) Regrowth of previously remitted lesions; (2) Newly added lymph nodes with any diameter >1.5cm; (3) New extranodal lesions with any diameter > 1.0cm; If <1.0cm, it needs to be clearly present and attributed to lymphoma; (4) Evaluable lesions of any size that can be directly attributed to lymphoma. |
|  | New lesion | New or recurrent FDG uptake lesions | New or recurrent bone marrow involvement |

**Appendix 2: NHL efficacy criteria (excluding PET-CT)**

| Curative effect | Physical examination | Lymph gland | Nodal mass | Bone marrow |
| --- | --- | --- | --- | --- |
| CR | Normal | Normal | Normal | Normal |
| CRU | Normal | Normal | Normal | Uncertain |
|  | Normal | Normal | Reduce>75% | Normal or uncertain |
| PR | Normal | Normal | Normal | Positive |
|  | Normal | Reduce≧50% | Reduce≧50% | Irrelevant |
|  | Liver/spleen reduce | Reduce≧50% | Reduce≧50% | Irrelevant |
| Relapse/PD | Liver/spleen enlargement  New lesion | New lesions or enlargement | New lesions or enlargement | Recurrence |

**Appendix 3: NHL efficacy criteria (including PET-CT)**

| Response | Definition | Nodular mass | Liver and spleen | Bone marrow |
| --- | --- | --- | --- | --- |
| CR | All evidence of the lesions is disappeared. | a) High affinity for FDG or positive for PET before treatment; PET negative lymph nodes of any size;  b) FDG affinity is uncertain or PET is negative, and the lesion shrinks to normal size on CT. | Can't be touched. The nodules disappear. | Repeated biopsy results were negative. If morphology can't confirm the diagnosis we need a negative immunohistochemical result. |
| PR | Measurable reduction of lesions, no new lesions | SPD in the 6 largest lesions decreased by ≥50% the size of other nodules did not increase  a) High affinity for FDG or positive for PET before treatment; There were 1 or more PET-positive lesions at the original affected site;  b) Uncertain FDG affinity or negative PET; CT showed focal shrinkage. | Reduction of nodule SPD (or maximum transverse diameter of a single nodule) ≥50%; The liver and spleen are not enlarged. | If it is positive before treatment, it will not be used as a criterion for judging efficacy. The cell type should be clear. |
| SD | CR/PR or PD not achieved | a) High affinity for FDG or positive for PET before treatment; The original lesion was still positive for PET after treatment. CT or PET shows no new lesions;  b) Uncertain FDG affinity or negative PET; CT showed no change in the size of the original lesion. | / | / |
| Relapse/PD | Any newly added lesion or original lesion increased in diameter by ≥50% | Any new lesions with diameters >1.5 cm appear; Multiple lesions had SPD increases of ≥50% or single lesions with short diameter >1 cm before treatment had maximum diameter increases of ≥50%. The lesions with high affinity for FDG or positive for PET before treatment were positive for PET after treatment. | SPD increased by >50% in all lesions | New and reissued |

Note: SPD/sum of the product of maximum vertical diameters 
